# Finding the right power balance: Better study design and collaboration can reduce dependence on statistical power

**DOI:** 10.1371/journal.pbio.3002423

**Published:** 2024-01-08

**Authors:** Shinichi Nakagawa, Malgorzata Lagisz, Yefeng Yang, Szymon M. Drobniak

**Affiliations:** 1 Evolution & Ecology Research Centre and School of Biological, Earth and Environmental Sciences, University of New South Wales, Sydney, Australia; 2 Theoretical Sciences Visiting Program, Okinawa Institute of Science and Technology Graduate University, Onna, Japan; 3 Institute of Environmental Sciences, Jagiellonian University, Kraków, Poland

## Abstract

Power analysis currently dominates sample size determination for experiments, particularly in grant and ethics applications. Yet, this focus could paradoxically result in suboptimal study design because publication biases towards studies with the largest effects can lead to the overestimation of effect sizes. In this Essay, we propose a paradigm shift towards better study designs that focus less on statistical power. We also advocate for (pre)registration and obligatory reporting of all results (regardless of statistical significance), better facilitation of team science and multi-institutional collaboration that incorporates heterogenization, and the use of prospective and living meta-analyses to generate generalizable results. Such changes could make science more effective and, potentially, more equitable, helping to cultivate better collaborations.

## Abstract 2

Statistical power analysis currently dominates experimental design. In this Essay, the authors argue that we should move away from the current focus on power analysis and instead encourage smaller scale studies and collaborative projects.

## Introduction

Given how much scientific progress has been made and how it is accelerating, it feels paradoxical to discover that >80% of research is potentially “wasted.” Two independent estimates from the fields of medicine and ecology confirm that this is the case [1,2]. The 2 primary sources of such waste are suboptimal study design and selective publication and reporting (the latter we refer to collectively as publication bias) [1–3]. Null hypothesis significance testing (NHST; Box 1), or more precisely, the misuse of NHST, may be the main culprit behind the issue of such publication bias because it makes the continuous nature of evidence artificially binary by using the threshold of *p*-values (α = 0.05) [4,5]. NHST facilitates not only selective publication and reporting but also *p*-hacking, HARKing (hypothesizing after results are known), and other types of what are known as “questionable research practices” [6,7]. Such misuses of NHST have been recently linked to massive failures to replicate published studies in many fields, the so-called “replication crisis” [8–10]. Indeed, researchers have been criticizing NHST for at least three-quarters of a century [11–13].

**Box 1. Glossary**

**Null hypothesis significance testing (NHST)**

In this framework, a null hypothesis is assumed (usually zero effect) for an intervention or phenomenon. After an experiment or observation, if the inferential statistic obtains a *p*-value of less than (or equal to) 0.05, the null hypothesis is rejected and the alternative hypothesis of nonzero effect is accepted (i.e., statistically significant or positive results). If a *p*-value higher than 0.05 is obtained, the null hypothesis is retained (i.e., nonsignificant or negative results).

***p*-hacking**

The NHST framework incentivizes *p*-values of less than or equal to 0.05. Therefore, arbitrary analytical decisions are often made to reach statistically significant results. For example, researchers might keep fitting different predictors (independent variables) to their statistical models until they produce a statistically significant result. *p*-hacking is one of the most common questionable research practices.

**HARKing**

The term represents an abbreviation for hypothesizing after results are known (HARKing). HARKing is a questionable research practice in which researchers generate a hypothesis to fit their known results so that they get positive results, which are easier to publish than negative results. A hypothesis should be created a priori.

**Linear mixed modeling**

It encompasses a group of statistical models with fixed effects and random effects, therefore often referred to as mixed-effects models. The model estimates regression coefficients for fixed effects, while it estimates variance components for random effects. The term “linear mixed(-effects) models” often indicate models assuming the Gaussian (normal) error structure but can include models with non-Gaussian errors, such as Poisson and binomial errors, which are often referred to as generalized linear mixed(-effects) models (GLMMs).

After decades of controversies and criticisms on NHST and *p*-values, it is somewhat surprising that concepts of statistical power and power analysis still seem to enjoy freedom from similar condemnations and disapprovals [14–16]. Power analysis is often used to determine a sufficient sample size necessary for *statistical significance*, thus fully endorsing NHST (see Box 2). Yet, at the same time, power analysis—when used correctly—improves study design by providing a sample size that gives more precise estimates (e.g., smaller standard error) [11,17]. Therefore, 2 powerful gatekeepers of academia, grant agencies, and ethics committees endorse and (at least indirectly) recommend power analysis for sample size calculations [18]. Their argument is that it is unethical and a waste of money to conduct underpowered or overpowered studies, leading to the recommendation of the nominal 80% statistical power. This argument seems undoubtedly true, especially for human trials [19,20]. Of relevance, researchers are often criticized for a lack of sufficient statistical power (or power analysis), not only in grant applications but also in scientific manuscripts, despite planning or doing the best they can within the constraints of time, finance, and other logistics.

**Box 2. Power analysis and related concepts**

Power analysis involves 4 parameters: statistical power, which is 1 minus a Type II error rate (1−*β*), often set to be 0.80; a Type I error rate, also known as significance level, *α*, usually fixed at 0.05; sample size, *N*; and standardized effect size, $E[\theta ]/\sqrt{Var[\theta ]}$, where *θ* is the effect size of interest and its population average (*E*[*θ*]) and variance (*Var*[*θ*]). If we know 3 of these 4, we can calculate the fourth unknown parameter.

Power analysis usually requires some estimates of standardized effect size (note that standardized mean difference *d* is an example of standardized effect size [21]). However, it is often challenging to obtain a good estimate, and published estimates are likely to be inflated [14,22,23]. It is interesting to note, when $E[\theta ]/\sqrt{Var[\theta ]}$ is examined, that there are 2 routes to having a large standardized effect size: either via having a large estimate of the population effect *E*[*θ*] or by having a small estimate of population variance *Var*[*θ*]. Indeed, in the vicious cycle of power analysis (see section on “The vicious cycle of publication bias and power analysis”), both are simultaneously happening, boosting the magnitude of the standardized effect size.

Assuming *α* = 0.05, (1−*β*) = 0.8, and the *d* values are as in the main text (e.g., *d* = 0.125), one can use the following formula to approximate the sample size required for 1 group of 2 independent sample groups [24]:

$$N=16\frac{Var[\theta ]}{E[\theta {]}^{2}}=16\frac{1}{d^{2}}.$$

In **S1 Supporting Information**, we provide an R script where we calculate the sample sizes used in the examples provided. Note that the above formula is incorrect for the interaction effect (e.g., sex difference in a treatment effect), as it involves 4 groups rather than 2, so in that scenario, one needs to use 32 instead of 16.

In this Essay, we challenge the premise that 80% statistical power is necessary for addressing many basic research questions (where a realistic study will almost always be underpowered yet worthwhile to conduct). We discuss how the misuse of power analysis contributes to research waste and the replication crisis in a nontrivial way and argue that undue focus on statistical power, similar to that on *p*-values, could counterintuitively encourage scientists to choose nonoptimal designs rather than improve study design. From the viewpoint of generalizability, we suggest that a set of several low-powered studies could be better than one high-powered study, even when the combined sample sizes are comparable in both scenarios [25,26]. Importantly, we discuss a series of potential alternatives and supplements to power analysis, which researchers and gatekeepers can implement. Our proposed paradigm shift can potentially improve science and its equity simultaneously by making science more collaborative.

## The vicious cycle of publication bias and power analysis

As already mentioned, one of the underlying causes of the replication crisis is publication bias, which is related to the filtering effect of NHST, causing an exaggeration of scientific evidence in terms of published effect sizes. Indeed, a series of large replication efforts have repeatedly shown that replication studies usually obtain much smaller effect sizes (e.g., 50% smaller [27]) than the original studies they sought to replicate [28–32]. In addition, recent meta-research studies have confirmed that inflated effect estimates in the published literature are common in many fields, including psychology, economics, ecology, and medicine [33–39]. For example, according to a meta-analysis of global change biology experiments that accounted for publication bias [37], a statistically significant effect reported in the literature is, on average, 2 to 3 times larger than a “true” effect. Furthermore, an average experiment in that field was severely underpowered (<40%) [35]. Therefore, published experiments often have small sample sizes, yet surprisingly large effects. The situation may be even worse for human randomized controlled trials (RCTs). A study found the median power of 23,551 RCTs to be only approximately 13% [23], probably because sample sizes were determined on the basis of inflated effects that had been previously reported.

**Fig 1:**
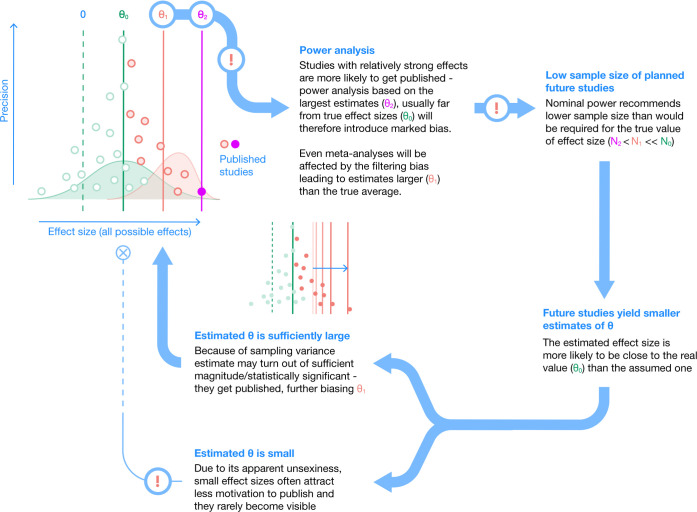
The vicious cycle of power analysis and publication bias. An example of how effect size *θ* can be inflated via selective publication and how power analysis, in its current use, can encourage this cycle to continue.

When deciding on a sample size for a new study, the most common method is to use a closely related published study or studies to generate an effect size estimate on which to base the power analysis [40]. But we know that published significant effects are inflated because of publication bias [14,22,23]. The consequence of using an overestimated effect in the power analysis is a sample size estimate that is far smaller than what is actually needed to “detect” a true effect [41]. Yet, sometimes, a value of *p* < 0.05 can be achieved by chance in this scenario, leading to the publication of yet another “inflated” effect, keeping this unfortunate and vicious cycle of power analysis going (also referred to as the winner’s curse [23,42,43]; see Fig 1).

We would argue that this cycle substantially contributes to research waste and the replication crisis. Inadvertently, grant agencies and ethics committees hold a key role in perpetuating this vicious cycle, as they endorse (and often require) power analysis. Some readers, particularly statisticians, may argue that this is not the fault of power analysis (and NHST) but of researchers who misuse it. However, given the prevalence of low statistical power in many studies, including RCTs [23], we believe that a critical rethink of how power analysis should be used or recommended is necessary.

## Two opposing forces

The current incentive structure and requirements of academia pull researchers in 2 completely opposite and incompatible directions: towards studies with small sample sizes (hereafter, small studies) and towards studies with large sample sizes (large studies). The prevalence of low-powered studies suggests that forces encouraging small studies are very strong. Research operates within the parameters of limited resources and time and a complex landscape of ethical regulation, all creating a huge incentive to conduct less costly experiments with small sample sizes. Such small studies will appear to have enough statistical power when designed under the expectation of a large effect size estimate, and researchers have no trouble finding such large, yet inflated, effect estimates in the literature. Resorting to meta-analytical estimates does not alleviate the issue. Although often more conservative (e.g., through active retrieval of unpublished estimates), such estimates are not free from publication bias and effect size inflation. Logistics aside, grant agencies usually appreciate the “value for money” offered by small studies, and ethics committees often prefer smaller to larger studies, thereby enabling researchers to maintain the vicious cycle together with grant and ethics boards.

By contrast, forces that encourage larger studies are present but often neglected. A study based on 13,322,754 abstracts from PubMed demonstrated that effect sizes declined between 1990 and 2015, while the frequency of statistically significant results increased, indicating the sample sizes of studies increased over the same period [44]. Academia pursues novelty, and such pursuits usually lead to the testing of more complex and subtle effects because the most obvious and large effects have usually already been discovered [14,45]. A case in point is gene–trait association studies where, in the early years, researchers were able to find genes with large effects, while more recently, such a discovery is rare; indeed, in recent years, most genome-wide association studies (GWASs) find many genes with small yet important effects [46]. It seems that most researchers nowadays are interested in research topics where the “true” effect is relatively small. Recent large-scale replication efforts have revealed that even effects believed to be large and general are usually small and too subtle to be useful or are even nonexistent, particularly in psychology [27–32]. It requires at least hundreds, if not thousands, of subjects to conduct an experiment that finds a significant yet small effect, which may be out of reach for many researchers.

Notably, our discussion has so far focused only on the main effect size in a study. To study interaction effects (e.g., sex differences in the treatment effect [47]), an 8-times larger sample size will be needed. This is the case when the interaction is the same magnitude as the main effect. A 16-times larger sample size is needed if it is assumed that the interaction is half of the main effect, which is more realistic [48]. Indeed, novel and important questions may often reside in interaction effects [49], which are usually smaller than the main effect size. Therefore, the implicit and explicit requirements of 80% power could stop researchers from exploring this frontier of knowledge. Such small effects relate to the idea that researchers should use the smallest effect size of interest for power analysis [50,51] (for an alternative, see [52]); however, using the smallest effect of interest often requires a larger study, which consequently requires more funding to perform (see **S1 Supporting Information**).

**Fig 2:**
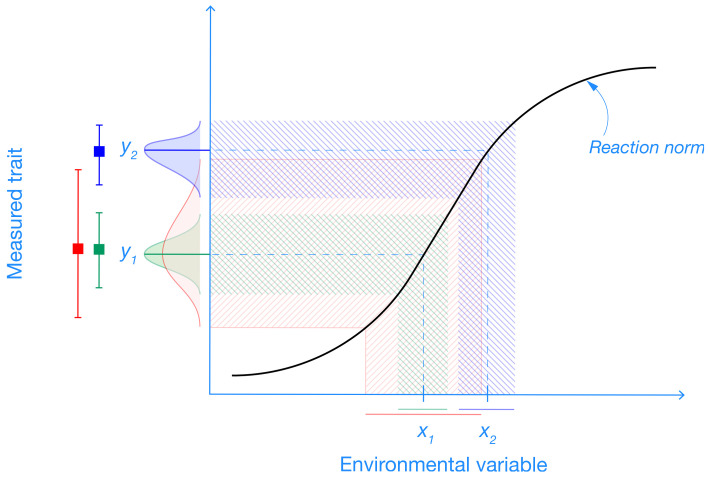
Plasticity of a trait in relation to an environmental variable. Traits are expressed differently (*y*_1_ and *y*_2_) depending on environmental conditions (*x*_1_ and x_2_). Therefore, excessive standardizations (of environments) will lead to unreplicable results. See Box 3 for the details of differently colored parts.

Of relevance, requirements for relatively large sample sizes (e.g., *N* = 100) would exclude many vertebrate researchers, particularly conservation biologists, from conducting their studies [53]. Furthermore, although labs that can afford large studies might manage to find a small, yet important effect, replicability and generalizability are far from guaranteed. If results are to be generalizable, experiments should include heterogenization, for example, by including different strains of animals and a range of environmental conditions [26,54,55] (Box 3). Thorough heterogenization necessarily increases within-subject variability, as it covers the landscape of different effect size magnitudes and variations (see Fig 2 in Box 3). So does heterogenization require an increase in sample size to maintain statistical power? Imagine that a researcher wants to heterogenize their 40 mice with regard to their strains. If they could get 20 different strains and create a complete block design by creating 20 blocks (i.e., each stain is assigned in both control and treatment groups), then they will not need to increase the sample size [54]. Yet, in reality, they are likely to get only 5 strains, creating replicates per control/treatment within the 5 blocks. In such a case, they do need to increase their sample size because mice within blocks (the same strains) are more similar to each other (i.e., not independent [54]). Taken together, how can the vicious cycle be escaped from, without also requiring a large sample size for many research questions? We argue that we must find and achieve a happy medium.

**Box 3. The importance of variability due to plasticity and heterogenization**

Variance observed in measured outcomes of empirical studies comes not only from between-individual variance and sampling error but also from environmental variance generated by the dependence of traits on external environmental variables (i.e., on the shape of a trait’s reaction norm [56–58]). Ignoring the reaction norm and forcing empirical studies (controlled experiments in particular) to eliminate all sources of environmental variation deemed “irrelevant” leads to increasingly irreplicable outcomes that simply explore different regions of a reaction norm mapping function [56,59] (Fig 2). Individual empirical studies focus on very specific environmental conditions to reduce unwanted variation in measured traits and amplify the expected differences (i.e., different points on the *x* axis in Fig 2). However, doing so in the presence of any meaningful relationship between the environment (*x*) and the measured trait (*y*) generates apparent discordance in observed phenotypes generated purely by their environmental plasticity.

If too much focus is given to maximizing statistical power (or precision), this process leads to an interesting paradox [56]. To measure traits as precisely as possible, individual studies generate more specific, nonoverlapping outcomes that hamper the reproducibility of key results. The solution is to rely less on a specific study and more on the comprehensive exploration of the underlying gradient of environmental variability [26,54,60]. In fact, less precise (e.g., lower powered) studies could paradoxically improve reproducibility as they generate outcomes that are not in conflict (note the overlap of the less precise blue density with 2 more precise and disconnected red and green densities on the *y* axis in Fig 2). Therefore, paying less attention to power analysis is only part of the solution. When coupled with a wider shift of the empirical paradigm (e.g., through heterogenization to represent whole ranges of underlying environmental and/or genetic variation in planned experiments [54,61,62]), we can move closer to resolving the ongoing reproducibility crisis.

## Better study design with less emphasis on power

The current focus on power will not help resolve the issue of 2 different forces acting on researchers. The best thing to do, therefore, is to shift attention to generating a better study design without worrying too much about reaching the nominal statistical power of 80% (apart from situations where large effects are expected, such as with pharmacological and toxicological interventions). We suggest using the AHARP (as high as reasonably practicable) principle, mirroring the ALARP (as low as reasonably practicable) principle, which is used in health and safety [63]. The AHARP principle assumes that it is often impossible to achieve enough power in a study when small effects and generalizations are considered. This principle aims to attain the best possible power or precision for a study within the constraints of budget and resources so that everybody (no matter their financial situation) can participate in research activities. Such a principle could mean that studies can have a relatively small sample size and be underpowered (e.g., *N* < 100). It is already known that small studies produce imprecise results [33], yet it is important to realize that, collectively, small studies themselves are unbiased; in other words, averaging results from many small studies could provide an accurate estimate of a “true” effect (see **S1 Supporting Information**). It is the filtering effect of the publication process on the basis of statistical significance (Fig 1) or other related criteria that produce exaggerated effect sizes, thereby making science unreliable.

Importantly, when we say “less emphasis on power” or emphasize the AHARP principle, this does not mean we think that the power (or precision) of studies should be ignored altogether. We only suggest that well-conceptualized study plans should not be cast aside because they fail to reach the expected >80% power. Thus, the AHARP principle does not equate to “free-for-all” research, and we would remind researchers that there are other aspects of study design to consider beyond just increasing sample size to improve study power and precision [64]. However, covering all aspects of study design is beyond the scope of this Essay (for further discussions, see [65,66]).

In many cases, statistical power can be improved by explicitly incorporating correlated structures between treatment and control groups, compared to using independent subjects alone (e.g., using sibling pairs as a complete block design; see **S1 Supporting Information**). By contrast, nested or hierarchical structures (e.g., siblings within mothers or animals within strains) could reduce power when such structures are statistically accounted for (if such structures are not accounted for, it is known as pseudoreplication [67–69]). Such correlated, nested, or hierarchical structures can be explicitly modelled using a (generalized) linear mixed modelling approach [70,71]. However, such complex designs pose difficulties for estimating precision and conducting power analysis. This is because the conventional algebraic formulas (Box 2) cannot be used to estimate the necessary sample size, so simulation must be used instead, which can become very complex [72–74]. One of the reasons for the difficulties is the necessity of knowing how correlated the data from a cluster is (e.g., how similar pups from the same mother are for a given measurement; see **S1 Supporting Information**). Nevertheless, researchers should be aware of the uses of correlated samples and that modelling correctly can provide a more precise and higher-powered design.

Researchers can also try to increase the precision of their measurements. For example, it is becoming increasingly easier to measure behavioral traits more precisely with AI-assisted video recording analyses [75]. Although not easy and potentially time-consuming, researchers could choose to optimize their study design, including improving their sampling strategies and using more precise measurement techniques, rather than relying upon power analysis, the correct implementation of which is often very difficult. Once they have their “best” sampling design regardless of its power, researchers may want to conduct a “design analysis,” which is defined as “a set of statistical calculations about what could happen under hypothetical replication of a study—that focuses on estimates and uncertainties rather than on statistical significance” [14] (see also [10]). The main part of the design analysis is calculating Type S (sign) error (the probability of getting the sign wrong when a result is statistically significant) and Type M (magnitude) error (the degree to which an effect is overestimated when significant) [14,22]. Type S and Type M errors are also defined in terms of statistical significance, but these 2 types of errors focus on estimates rather than significance [14].

To make our position clear, we think the concepts of statistical significance and power, along with *p*-values and power analysis, are important for navigating the scientific literature and, when used correctly, can be useful [76,77]. However, we feel that grant agencies (including grant assessors) and ethics committees should be satisfied if researchers have done due diligence when coming up with the best study design. If researchers can report their study design’s Type S and Type M error rates, we believe this would provide a better benchmark for a proposed empirical project than conventional power analysis: We would even encourage researchers to report statistical power along with Type S and Type M error rates.

## From vicious cycle to virtuous cycle

**Fig 3:**
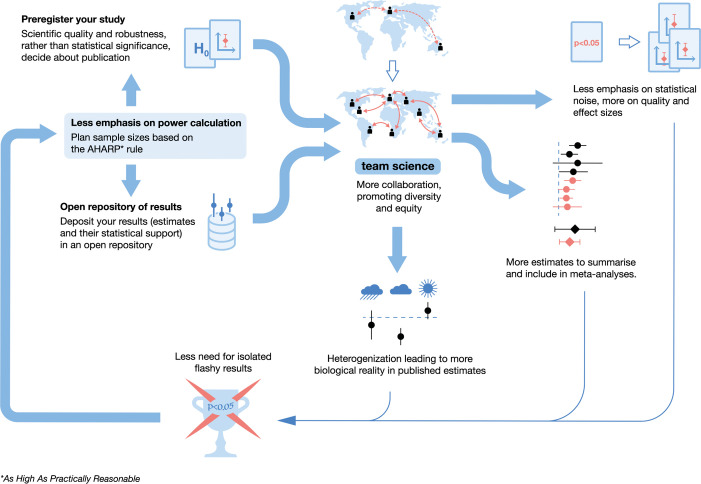
The virtuous cycle of research. A visualization of how our proposed paradigm shift could start a virtuous cycle that empowers researchers and better science.

We believe that grant and ethics bodies should not always expect researchers to determine sample size via power analysis. As argued above, such usage of power analysis might influence researchers to choose suboptimal study designs and could maintain the vicious cycle of biased research findings and research waste (Fig 1). Instead, researchers, grant agencies, and ethics boards could be working together to turn the vicious cycle into a virtuous cycle (Fig 3).

### Registration and full reporting

Power analysis, used wrongly, could eliminate interesting research ideas that could otherwise, in accumulation, contribute to a field. Instead, grant agencies could ask researchers to (pre)register their funded and approved studies (note that the terms “registration” and “preregistration” are used interchangeably for the same process [78]) and publish their work regardless of the statistical significance of the results (Fig 3). We propose that funders and journals team up to ensure that all registered studies are published, regardless of their results. According to some estimates, more than 50% of studies remain unpublished, mainly because the results did not reach statistical significance [2,79]. Registration, along with registered reports, can partially mitigate this issue [80]. Relatedly, there are novel ways of disseminating research, such as Octopus and ResearchEquals, both of which allow different components of research to be published in a separate yet modular manner (e.g., hypothesis, method, result, code).

Unfortunately, we do not think that registration and these related innovations will be the main solution for publishing negative results. For years, scientists have repeatedly argued, with little effect, that a study needs to be published regardless of statistical significance, yet it seems that much research remains unpublished [2,79]. This is not surprising because proper incentives for doing so are not yet in place. Therefore, we propose that a free repository of statistically nonsignificant results (or all results) be created, preferably associated with study registration. In this repository, one could fill in study results using a template in a short amount of time, making the data findable, accessible, interoperable, and reusable (FAIR) [81]. Setting up such a repository, and mandating its use, is exactly what grant agencies and ethics committees could be doing. Archiving nonstatistically significant results is essential because results from well-designed studies are unbiased regardless of statistical power or how small a study was. Such a repository would enable the community to access the results of all relevant studies for later syntheses. Reporting results to registries is mandatory for some medical RCTs, although there seem to be some issues with compliance [82,83]. Grant agencies and ethics committees could certainly help fix such compliance issues [84].

### Collaboration to improve reproducibility and equity

Pluralism and diversity make science better [85,86]. In addition to needing greater pluralism, we need to realize that what one study can achieve is limited, however powerful, well-designed, and expensive such a study might be [25,87,88]. Grant agencies and ethics committees therefore have an important role in fostering and supporting collaboration for multiple studies (Fig 3).

If grant agencies and ethics committees allowed AHARP study designs, science could move towards becoming more equitable, diverse, and inclusive (EDI) [86,89]. For many emerging questions where large effects are not expected, only those with sufficient funding are able to conduct the large experiments that power analysis would demand. However, being inclusive of any studies, regardless of their power, would encourage more research from different institutions across the globe. Of importance, a simulation study indicates that even well-funded laboratories should consider conducting several low-powered studies (e.g., 30% power) rather than a single high-powered study (80% power; note that the latter is approximately 4 times larger than the former [25]). This is because when the effect of interest has a realistic amount of heterogeneity (e.g., due to meaningful temporal and locational variation), a single high-powered study has a higher Type I error rate than an aggregation of several low-powered studies, which can better accommodate heterogeneity [25]. Therefore, even the well-funded would do well to collaborate with others at different institutions to make their experimental results more robust and in line with the idea of heterogenization (Box 3). Such designs not only improve the overall power of estimates but also make them more biologically relevant and generalizable. Grant agencies, along with ethics committees, could encourage and specifically fund multi-institutional experiments, through which they could provide more opportunities to researchers from traditionally marginalized groups, spreading EDI in science [86,89] (for a related example of when and how such an experiment could be funded, see [90]). Such a multi-institutional experiment, combined with a later synthesis, can be seen as a “prospective” meta-analysis [91].

Indeed, this type of synthesis is exactly what big team science projects have done and are trying to do. In recent years, CERN-style, big team science projects have emerged and spread across many fields [92]. Examples include ManyBabies [93], the Reproducibility Project: Cancer Biology [30], SPI-Birds [94], and the Nutrient Network [95], (see also [96] for an example of how citizen science can be harnessed to increase statistical power and precision). Such team science projects form a collaborative community across several institutions to conduct a prospective meta-analysis, which resolves the post hoc nature of traditional meta-analyses. Not surprisingly, many post hoc meta-analytic estimates are also much larger than those from multilaboratory replication efforts [32] (e.g., Many Labs [97,98]). This result indicates that meta-analytic means are often overestimated, although bias-corrections of meta-analytic mean estimates are possible and can be effective [99]. Therefore, we propose a shift from traditional to prospective meta-analyses.

Big team science projects are able to do more than just produce a prospective meta-analysis because of the communities they create. Such communities can organize a meta-analysis to be continuously updated (i.e., a living synthesis) [100], which has recently been described as an “open synthesis community” [101]. Notably, team science is not without its problems; for example, there are concerns regarding how to fairly credit each scientist involved and whether team science could increase inequity rather than decrease it [92,102,103]). But this is where grant agencies could intervene to introduce new criteria for recognizing scientific contributions and make sure large collaborative efforts, which they fund, address EDI fully [87]. Regardless, it will require coordination among researchers, funders, institutions, and other relevant committees and organizations (e.g., learned societies) to make team scientific activities easier and fair [104].

## Conclusions

We began this article by referring to 2 major causes of “research waste”: suboptimal study design and publication bias (selective publication and reporting). We have argued that, although power analysis helps study design in theory, paying less attention to statistical power may improve study design in practice, just like paying less attention to statistical significance (threshold *p*-values) could alleviate the issue of publication bias. Hopefully, we have convinced many, especially those on grant and ethics committees, that it is time for a paradigm shift in our approach to research. We must encourage better study designs with less focus on power; (pre)registration and full publication of all data; team science or multi-institutional collaborations that allow realistic incorporation of heterogenization; and prospective and living meta-analyses to reach generalizable results. By adopting those changes, we can break out of the vicious cycle into the virtuous cycle (Fig 3). In such a virtuous cycle, less emphasis on statistical power could start and maintain a more collaborative, equitable, and diverse scientific environment, where both underestimates and overestimates are welcome and integrated to achieve an estimate closer to a “true” effect. To get there, we need to find the right “power” balance.

## Supporting information

S1 Supporting Information An HTML file containing 3 sections: Section 1, a fictitious story providing different experimental scenarios using mice; Section 2, calculating statistical power under the scenarios introduced under Section 2; and Section 3, showing how small low-powered studies can be aggregated via a meta-analysis. (HTML)

## Abbreviations

AHARP: as high as reasonably practicable
ALARP: as low as reasonably practicable
EDI: equitable, diverse, and inclusive
FAIR: findable, accessible, interoperable, and reusable
GLMM: generalized linear mixed(-effects) model
GWAS: genome-wide association study
HARKing: hypothesizing after results are known
NHST: null hypothesis significance testing
RCT: randomized controlled trial
