# Supplementary material for: Finding the right power balance: Better study design and collaboration can reduce dependence on statistical power
Source: PLoS Biol. 2024 Jan 8;22(1):e3002423. doi: 10.1371/journal.pbio.3002423 (PMC10773938; doi:10.1371/journal.pbio.3002423)
Supplement: S1 Supporting Information — (HTML) [file pbio.3002423.s001.html]

Finding the right power balance: better study design and collaboration can reduce dependence on statistical power


Code 

- Show All Code
- Hide All Code
- Download Rmd

# Finding the right power balance: better study design and collaboration can reduce dependence on statistical power

# Shinichi Nakagawa, Malgorzata Lagisz, Yefeng Yang & Szymon Drobniak

## Finding the right power balance: better study design and collaboration can reduce dependence on statistical power

- Electronic
  Supplementary Material
- Setups
- Aims of this
  Supporting Information
- Section 1: A Fiction
  - A likely story
    of a principal investigator
- Section 2: Power
  Calcuation
  - Preambles
  - Estimating
    sample sizes in the ‘fictitious’ experiment with large
    effects
  - Estimating
    sample sizes in the ‘ficticious’ experiment with realstic (small)
    effects
  - Corrleated
    samples and statistical power
- Section 3: Small N &
  Meta-analysis
  - Simulating the
    population
  - Simulation of multiple
    studies
  - Scenario 1
    - low-powered studies are excluded
  - Scenario
    2 - conservative publication of all estimates
  - If
    power calculation is based on meta-analytical
    effect-sizes
- References
- R Session Information

29
November 2023

# Electronic Supplementary Material

# Setups

Loading packages and custom functions. If your computer do not have
the required packages, please install them via
`install.packages("package.name")`

```
# load required packages
pacman::p_load(dplyr, magrittr, tidyr, stringr, ggplot2, cowplot,
    patchwork, tidyverse, here, readxl, retrodesign, pwr, Superpower,
    pander, metafor)

# custom function for approximate sample size for main
# effect and interactive effect
short_cut <- function(d, method = c("normal", "interaction")) {
    method <- match.arg(method)
    if (method == "normal") {
        size <- 16 * (1/d^2)
    } else {
        size <- 32 * (1/d^2)
    }
    size
}
```

# Aims of this Supporting Information

There are three different sections in this document: 1) we presents
an fictional story that include some of examples of small effects mainly
focusing on interaction effects we mention in the main text (A Fiction),
2) we show how we obtain sample sizes presented in this fictitious story
(Power Calculation) and 3) we show why it is important to publish
regardless of sample sizes or the power of studies (Small N &
Meta-analysis)

# Section 1: A Fiction

## A likely story of a principal investigator

Imagine that you are a new principal investigator, PI, studying the
detrimental consequences of obesity on cognition using a mouse model.
You must put together your first grant as a lead PI. So you start with
power analysis to determine sample size for a new idea on investigating
the effect of maternal obesogenic diet on offspring’s memory tasks in
mice and sex-specific differences in the tasks (Fig S1A). You need an
estimated effect of the treatment and sex difference; of course, nobody
has yet performed such a study, so you do not know an exact effect size.
An earlier study examining maternal obesogenic diet on offspring’s
cognition, using a different assay, has suggested surprisingly large
effects: a 30% increase in the number of errors in males and 20% in
females (i.e., 10% difference between the sexes; standardized mean
difference, \(d\) = 1.0, 0.67, 0.33,
for male and female treatment effects, and the sex difference,
respectively). Using these effects sizes, power analysis, which assumes
the independence of samples, shows you will need the sample sizes of 16
male and 36 female mice (F1 generation) in each control and treatment
groups (32 males and 72 females in total) to detect a similar effect in
your proposed experiment. So, you decide that you will use 72 offspring
mice for each sex to have a nicely balanced design (Fig S1B). But then,
you will need at least 36 x 2 mother mice (F0 generation) considering
the independence of samples at least in treatment vs control groups
(i.e., taking one male and one female pup from each mother for each
group; see Fig S1C).

### Figure S1

An experimental design with hypothetical effects. Panel A shows an
overview of experimental design with four groups: 1) control females
(Ncf), 2) treatment females (Nof), 3) control males (Ncm) and 4)
treatment males (Nom) with N representing ‘sample size’. Panel B shows
assumed effect sizes as % and d (standardized mean difference; assuming,
e.g. the baseline mean of 100 and standard deviation of 20) and
resultant sample sizes from power analysis. Panel C shows two possible
scenarios based on power analysis: F0 are mothers, F1 are offspring
females (pinkish) and males (navy) with a target sample size of 36
offspring per sex per group.

Although this planned experiment is just one part of your grant
proposal, it is already eating up the budget, even without considering
sex differences. Thus, you decide that it is acceptable to take 4 male
and 4 female pups from each mouse mother, so you only need 9 x 2 (18)
mothers in total (Fig S1C). But your design now violates the statistical
assumption of independent samples, used for the power analysis (see Fig
1). By the way, what you decide not to mention in your grant proposal is
that the original power analysis also suggest you would need 294
independent offspring of each sex per group to compare sex difference in
treatment effect, or statistically speaking, testing for an interaction
among the four groups (i.e., 1,176 offspring mice from 294 x 2 [588]
mothers; for details). This decision of concealment, despite your
interest in sex difference, is because not only would incorporating such
a design be far too expensive, but also running such an experiment would
be too labor intensive, even with a PhD student and technician. Surely,
the ethics committee would not like such a large mice experiment even if
you got funded.

Now let’s use smaller and more realistic, albeit, hypothetical,
estimates of effect sizes: 5%, 3%, 2% difference (\(d\) = 0.16, 0.1 or 0.06, respectively) for
the treatment effect for males, females, and the sex difference (note
that replication work suggests these hypothetical effect sizes are
reasonable). For this scenario, you will need 574 x 2 (1,148) males,
1,600 x 2 (3,200) females and 7,128 offspring mice in each of the
control and treatment groups of both sexes (i.e., 28,512 mice in total)
to reach statistical significance. You must not forget that you will
also need 7,128 x 2 (14,256) mothers to produce these offspring mice
(only using one male and one female pup per mother to meet the
independence assumption). There is no way of doing such a
‘bigger-than-your-university-facility-can-take’ experiment, especially
as a new PI (at least, not by yourself). In fact, there is little or no
incentive whatsoever to provide realistic estimates of sample sizes when
grant bodies request ‘value for money’ and ethics committees ask you to
minimize the use of animals. Instead, such well-intended recommendations
have been incentivizing scientists to use the smallest sample sizes
possible based on greatly inflated published effect sizes (see the main
text). Note this example was inspired by our earlier meta-analyses of
related studies (Lagisz et al. 2014, 2015; Anwer et al. 2022)

# Section 2: Power Calcuation

## Preambles

Statistical power are determined by the following three
parameters:

1. Type I error probability, \(\alpha\), also known as significance
   threshold, which is usually fixed at 0.05 (see Table I);
2. sample size, \(n\), that is the
   number of subjects required for an experiment
3. standardized effect size, \(E[\theta]/\sqrt{Var[\theta]}\), where \(\theta\) is the effect size of interest,
   which is indicated by the real difference between two groups (in our
   case: obesogenic diet vs. control diet), \(E[\theta]\) is the population
   average/expectation, and \(Var[\theta]\) is the respective variance;
   note that standardized mean difference \(d\) is an example of a standardized effect
   size (for more on effect size, see also Fig 2 and Box 2).

```
# drawing Fig 1 Parameter 1: alpha level vs. power

#### set a range of alpha levels (0.01 to 0.1)
alpha_range <- seq(0.001, 1, by = 0.01)

#### calculate power at the set alpha levels using a medium
#### magnitude of standardized effect size 0.5 with a
#### standard deviation of 0.2
power_range <- retro_design(A = 0.5, s = 0.2, alpha = alpha_range)

#### create a data frame
power_vs_alpha <- data.frame(alpha = alpha_range, power = power_range$power)

#### plot
power_vs_alpha_plot <- ggplot(power_vs_alpha) + geom_line(aes(x = alpha,
    y = power), show.legend = F) + scale_y_continuous(breaks = seq(0,
    1, 0.2), limits = c(0, 1)) + geom_hline(yintercept = 0.8,
    colour = "red") + labs(x = "Type 1 error (alpha)", y = "Statistical power",
    title = "(A) alpha level vs. power") + theme_bw()


### Parameter 2: n vs. power

#### set a range of n (2 to 100)
n_range <- seq(2, 100, by = 2)

## calculate power for a two-sample t test
## (two-independent-samples-design) using a medium
## magnitude of standardized effect size 0.5
power_range2 <- pwr.t.test(d = 0.5, n = n_range, sig.level = 0.05,
    type = "two.sample", alternative = "two.sided")

#### create a dataframe
power_vs_n <- data.frame(n = n_range, power = power_range2$power)

#### plot
power_vs_n_plot <- ggplot(power_vs_n) + geom_line(aes(x = n,
    y = power), show.legend = F) + scale_y_continuous(breaks = seq(0,
    1, 0.2), limits = c(0, 1)) + geom_hline(yintercept = 0.8,
    colour = "red") + labs(x = "Sample size (n)", y = "Statistical power",
    title = "(B) n vs. power") + theme_bw()

### Parameter 3: effect size vs. power

#### create a plausible range of standardized effect sizes
es_range <- seq(0.01, 1.01, by = 0.01)

#### calculate power with alpha 0.05
power_range3 <- retrodesign::retro_design(A = es_range, s = 0.2,
    alpha = 0.05)

#### create a dataframe
power_vs_es <- data.frame(es = es_range, power = power_range3$power,
    alpha = rep(c("0.05"), length(es_range)))

#### plot
power_vs_es_plot <- ggplot(power_vs_es) + geom_line(aes(x = es,
    y = power), show.legend = F) + scale_y_continuous(breaks = seq(0,
    1, 0.2), limits = c(0, 1)) + geom_hline(yintercept = 0.8,
    colour = "red") + labs(x = "Effect size (d)", y = "Statistical power",
    title = "(C) effect size vs. power") + theme_bw()


### put all figures together
power_plot <- power_vs_alpha_plot/power_vs_n_plot/power_vs_es_plot
power_plot
```

### Figure S2

An example showing how the three parameters affect the statistical
power: (A) Type I error (\(\alpha\)),
(B) Sample size (\(n\)), (C) Magnitude
of the standardized effect size (\(d\)). These figures are simulated using
`retro_design()` function in `retrodesign` package
(Gelman & Carlin 2014). See the corresponding code chunk for
detailed code.

From Figure S2A we can see that when an experiment commits a higher
Type 1 error (which we do not want), it is easier to achieve a desired
statistical power (i.e., Cohen’s recommendation: 80% power). Increasing
sample size (\(n\)) and magnitude of
standardized effect size are effective ways to increase the statistical
power of a given experiment (Figure S2B and S2C).

## Estimating sample sizes in the ‘fictitious’ experiment with large effects

When designing an ‘fictitious’ diet experiment in your grant
proposal, You choose a common significance threshold, \(\alpha\) = 0.05 and the nominal power level
of 80%. Based on your pilot or external information (e.g., relevant
studies or a meta-analysis on maternal effect), you assume maternal
obesogenic diet will lead to a 30% increase in the number of mistakes in
a memory task in males, 20% in females and 10% difference between the
sexes. To quantify the diet effect using a standardized effect size
(i.e., \(d\)). We assumed the
followings:

control group (both male and female) - mean = 100 (arbitrary unit)
and standard deviation (sd) = 30;

male treatment group - mean = 130 and sd = 30;

female treatment group - mean = 120 and sd = 30.

Note that we assume the homogeneity of variances among groups
(i.e. sd = 30).

```
## scenario 1: large effects

### set up an independent design
design <- ANOVA_design(
  design = "2b*2b", # independent design, which means no correlation
  n =290, # the sample size in each group for testing sex difference
  mu = c(130, 120, 100, 100), 
  sd = 30,
  labelnames = c("diet", "obesogenic ", "control ", "sex", "male", "female"),
  plot = FALSE)

meanplot_largeES <- design$meansplot + labs(x = "Groups", y = "Mean")
Figure_S3 <- meanplot_largeES
Figure_S3
```

### Figure S3

Visualization of the assumed means and standard deviation (sd) of
each group, using the package `Superpower`. Error bars
represent sd. Here we assume each group is independent (note that this
is not quite true if we take one male and one female from one mother but
for convenience, let’s assume this)

### Presumed effect sizes (large)

Using these means and sds, we have the following standardized mean
difference \(d\), corresponding %
differences:

1. 1.0, corresponding to a 30% increase in the number of mistakes in
   a memory task in males after a diet intervention;
2. 0.67, corresponding to a 20% increase in the number of mistakes
   in a memory task in females after a diet intervention;
3. 0.33, corresponding to a 10% sex difference or interaction
   between diet and sex.

You are planning to use a typical two-sample t-test to examine the
statistical significance of the diet effect in males and females. Then
you can approximate sample size required for each group using the
following formula (Lehr 1992):

\[
n = 16 \frac{Var[\theta]} {E[\theta]^2} = \text{16} \frac{1} {d^2}
\] However, for the last effect (interaction effect), this
requires comparing 4 groups so that this formula does not work. Yet you
could still use this formula by replacing 16 with 32 as interaction
involves four groups rather than two.

### Sample size calculation for diet effects

In the following section, we use our custom function (based on the
above formula) and one existing R package `pwr` to estimate
the sample size used in your proposed experiment with different
scenarios (sample size mentioned in the fictitious story in the main
text).

```
# Cohen's d male
(130 - 100)/30
```

```
## [1] 1
```

```
# Cohen's d female
(120 - 100)/30
```

```
## [1] 0.6666667
```

```
# the first set (surprising large effects)

## male treatment effect
pwr.t.test(d = 1, sig.level = 0.05, power = 0.8, type = "two.sample",
    alternative = "two.sided")
```

```
## 
##      Two-sample t test power calculation 
## 
##               n = 16.71472
##               d = 1
##       sig.level = 0.05
##           power = 0.8
##     alternative = two.sided
## 
## NOTE: n is number in *each* group
```

```
# our result from our custom function is very close
pwr_independent_m_d1 <- short_cut(d = 1, method = "normal")

## female treatment effect
pwr.t.test(d = 0.67, sig.level = 0.05, power = 0.8, type = "two.sample",
    alternative = "two.sided")
```

```
## 
##      Two-sample t test power calculation 
## 
##               n = 35.95537
##               d = 0.67
##       sig.level = 0.05
##           power = 0.8
##     alternative = two.sided
## 
## NOTE: n is number in *each* group
```

```
pwr_independent_f_d0.67 <- short_cut(d = 0.67, method = "normal")
```

### Sample size calculation for sex difference (interaction)

We cannot use `pwr.t.test` for getting sample size for the
sex difference. So first we can use our formula and then, we use the
package `Superpower` to obtain a simulation based sample
size.

```
# Cohen's d sex difference (interaction)
((130 - 100) - (120 - 100))/30
```

```
## [1] 0.3333333
```

```
pwr_independent_i_d0.33 <- short_cut(d = 0.33, method = "interaction")
pwr_independent_i_d0.33
```

```
## [1] 293.8476
```

Main results of the outputs of the `ANOVA_exact` when
detecting large effects:

```
## scenario 1: large effects perform ANOVA and calculate
## power for in dependent design
power_results <- ANOVA_exact(design, alpha_level = 0.05, verbose = FALSE)

power_results$main_results
```

```
##              power partial_eta_squared    cohen_f non_centrality
## diet     100.00000          0.14836492 0.41738692     201.388889
## sex       80.94609          0.00692025 0.08347738       8.055556
## diet:sex  80.94609          0.00692025 0.08347738       8.055556
```

Simulation (using the `ANOVA_exact` function) shows that
collecting data from \(n\) = 294 F1
mice (per group) has 81% power for the interaction or sex difference
(see code chunk for R syntax).

We also plot a power curve over a range of sample sizes (Figure S4),
from which you can visually explore whether the expected power is
achieved for the interaction (bottom panel), and if so, at which sample
size.

```
plot.power <- plot_power(design, min_n = 5, max_n = 300, desired_power = 80,
    plot = FALSE)
```

```
## Achieved Power and Sample Size for ANOVA-level effects
##   variable                  label   n achieved_power desired_power
## 1     diet Desired Power Achieved  12          80.60            80
## 2      sex Desired Power Achieved 284          80.13            80
## 3 diet:sex Desired Power Achieved 284          80.13            80
```

```
plot.power$plot_ANOVA + labs(x = "Sample size per group")
```

### Figure S4

Power curves for large inter-generational effect in a dependent
design (diet and sex are manipulated between animals). Top panel = the
main effect - diet; Middle panel = the main effect - sex; Bottom panel =
the interactive effect, sex difference. The orange horizontal lines
denote the expected statistical power (80%). Note that the main diet
effect is an average effect over the two sexes.

As you see, the simulation-based method suggests we need 284 subjects
to reach 80% to detect the interaction effect, which confirm what we
obtained form the formula was close enough.

## Estimating sample sizes in the ‘ficticious’ experiment with realstic (small) effects

This time, we assume maternal obesogenic diet will have more
realistic effects on pups’ memory: a 5% increase in males, 3% in females
and thus 1% difference in the diet effect between the sexes
(interaction).

To calculate \(d\), you assume:

control group (both male and female) - mean = 100 (arbitrary unit)
and sd = 30;

male treatment group - mean = 105 and sd = 30;

female treatment group - mean = 103 and sd = 30.

```
## scenario 2: small effects
### set up an independent design
design2 <- ANOVA_design(
  design = "2b*2b", # independent design
  n =7128, # the sample size used for testing sex difference
  mu = c(105, 103, 100, 100), 
  sd = 30,
  labelnames = c("diet", "obesogenic ", "control ", "sex", "male", "female"),
  plot = FALSE)

meanplot_smallES <- design2$meansplot + 
  labs(x = "Groups", y = "Mean", title = "Realistic small effect")
meanplot_smallES
```

### Figure S5

Visualization of the expected means and standard deviation (sd) of
each group using the package `Superpower` under more
realistic scenarios. Error bars represent sd. Here we assume each group
is independent (note that this is not quite true if we take one male and
one female from one mother but for convenience, let’s assume this).

### Presumed effect sizes (small)

As with the above, we assumed all groups share a common sd = 30
(population standard deviation). Then you can obtain the following
standardized mean difference \(d\):

1. 0.16, corresponding to 5% increase in the number of mistakes in a
   memory task in males after a diet intervention;
2. 0.1, corresponding to 3% increase in the number of mistakes in a
   memory task in females after a diet intervention;
3. 0.06, corresponding to 2% sex difference or interaction between
   diet and sex.

### Sample size calculation for diet effects

Following similar procedures in estimating sample sizes for large
effects (see above), you can obtain sample sizes with these new presumed
effect sizes

```
# Cohen's d male
(105 - 100)/30
```

```
## [1] 0.1666667
```

```
# Cohen's d female
(103 - 100)/30
```

```
## [1] 0.1
```

```
# the first set (realistic small effects)

## male treatment effect
pwr.t.test(d = 0.167, sig.level = 0.05, power = 0.8, type = "two.sample",
    alternative = "two.sided")
```

```
## 
##      Two-sample t test power calculation 
## 
##               n = 563.8262
##               d = 0.167
##       sig.level = 0.05
##           power = 0.8
##     alternative = two.sided
## 
## NOTE: n is number in *each* group
```

```
pwr_independent_m_d0.167 <- short_cut(d = 0.167, method = "normal")

## female treatment effect
pwr.t.test(d = 0.1, sig.level = 0.05, power = 0.8, type = "two.sample",
    alternative = "two.sided")
```

```
## 
##      Two-sample t test power calculation 
## 
##               n = 1570.733
##               d = 0.1
##       sig.level = 0.05
##           power = 0.8
##     alternative = two.sided
## 
## NOTE: n is number in *each* group
```

```
pwr_independent_f_d0.1 <- short_cut(d = 0.1, method = "normal")
```

### Sample size calculation for sex difference (interaction)

We can also use our formula to estimate (assuming independence of all
groups)

```
# Cohen's d sex difference (interaction)
((105 - 100) - (102 - 100))/30
```

```
## [1] 0.1
```

```
# sex difference
pwr_independent_i_d0.067 <- short_cut(d = 0.067, method = "interaction")
pwr_independent_i_d0.067
```

```
## [1] 7128.536
```

We use a similar simulation-based approach to empirically calculate
power for the interaction effect using `Superpower`. Main
results of the outputs of the `ANOVA_exact` when assuming
small effects:

```
## scenario 2: small effects perform ANOVA and calculate
## power for in dependent design
power_results2 <- ANOVA_exact(design2, alpha_level = 0.05, verbose = FALSE)
power_results2$main_results
```

```
##              power partial_eta_squared    cohen_f non_centrality
## diet     100.00000        0.0044253969 0.06667134         126.72
## sex       80.35012        0.0002777396 0.01666784           7.92
## diet:sex  80.35012        0.0002777396 0.01666784           7.92
```

Simulations (using the `ANOVA_exact` function) show that
collecting data from \(n\) = 7129 F1
mice (per group) has 80.35% power for the interaction or sex difference.
So the simulation result seems to catch with the sample size estimated
by the formula.

## Corrleated samples and statistical power

As mentioned, correlated samples can increase the statistical power
of an experiment so that we require fewer samples. Here, we assume that
sibling traits are correlated (\(r\) =
0.5) regardless of sex. We find \(n\) =
3564 can reach the expected statistical power (80%) for interaction
(i.e., sex difference). This number (3567) corresponds to

```
## scenario 1: small effects
### perform ANOVA and calculate power for in independent design
### assuming the siblings are very similar to each other - r = 0.5
design3 <- ANOVA_design(
  design = "2w*2w", # dependent design 
  n =3564, 
  r = 0.5,
  mu = c(105, 103, 100, 100), 
  sd = 30,
  labelnames = c("diet", "obesogenic", "control", "sex", "male", "female"),
  plot = FALSE)

power_results3 <- ANOVA_exact(design3, alpha_level = 0.05, verbose = FALSE)
power_results3$main_results
```

```
##              power partial_eta_squared    cohen_f non_centrality
## diet     100.00000         0.034344069 0.18858827         126.72
## sex       80.33173         0.002217916 0.04714707           7.92
## diet:sex  80.33173         0.002217916 0.04714707           7.92
```

This number (3567) corresponds to the value calculated from the
following formula:

\[
n\_{interaction} = \frac{32} {d^2}(1 - r)
\]

Using this formula, we can assume a lower correlation (\(r\) = 0.25) and then, we find \(n\) = 5346. As before, we can verify this,
using `ANOVA_design`:

```
## scenario 2: small effects
### perform ANOVA and calculate power for in independent design
# assuming the siblings are very similar to each other - r = 0.25
design4 <- ANOVA_design(
  design = "2w*2w",   # dependent design 
  n =5346, 
  r = 0.25,
  mu = c(105, 103, 100, 100), 
  sd = 30,
  labelnames = c("diet", "obesogenic", "control", "sex", "male", "female"),
  plot = FALSE)

power_results4 <- ANOVA_exact(design4, alpha_level = 0.05, verbose = FALSE)
power_results4$main_results
```

```
##              power partial_eta_squared    cohen_f non_centrality
## diet     100.00000         0.023159080 0.15397447         126.72
## sex       80.33874         0.001479566 0.03849362           7.92
## diet:sex  80.33874         0.001479566 0.03849362           7.92
```

As you see, with \(n\) = 5346, we
have ~80% power. We note that, as mention in the text, for more complex
designs (e.g. including different strains of mice), we cannot use the
formula or the functions form `Superpower`. We need to use
other software packages which could accommodate such design
features.

# Section 3: Small N & Meta-analysis

## Simulating the population

Two groups of individuals, e.g. males and females, with means of 10
and 12, respectively. Variances are equal (= 100).

```
set.seed(7777)
mean_m <- 10
mean_f <- 20
sd <- sqrt(100)
N <- 888

males <- rnorm(N, mean_m, sd)
females <- rnorm(N, mean_f, sd)

popdata <- data.frame(y = c(males, females), sex = rep(c("m",
    "f"), each = N))
```

```
ggplot(data = popdata) + geom_density(aes(y = y, colour = sex,
    fill = sex), alpha = 0.4, linewidth = 0.8) + geom_boxplot(aes(x = 0,
    y = y, group = sex, colour = sex), position = "dodge2", width = 0.01,
    size = 0.8) + xlab("Density") + ylab("Measured trait") +
    theme_bw() + coord_flip()
```

### Figure S6

Visualizing the difference between females (f) and males (m).

## Simulation of multiple studies

Simulate M = 15 independent studies that look at the population,
testing for difference between males and females. Studies range from n =
5 to n = 10

```
M <- 15
samples <- 5:10
outdata <- data.frame(diff = numeric(0), t = numeric(0), p = numeric(0),
    n = numeric(0), pooled_s = numeric(0))

for (i in 1:M) {
    sample <- sample(samples, 1)
    males_s <- subset(popdata, sex == "m")[sample(1:N/2, size = sample,
        replace = F), ]
    females_s <- subset(popdata, sex == "f")[sample(1:N/2, size = sample,
        replace = F), ]

    expdata <- rbind(males_s, females_s)

    test <- t.test(y ~ sex, data = expdata)
    outdata[i, "diff"] <- test$estimate[1] - test$estimate[2]
    outdata[i, "n"] <- sample
    outdata[i, "t"] <- test$statistic
    outdata[i, "p"] <- test$p.value
    outdata[i, "pooled_s"] <- sqrt(((sample - 1) * var(males_s$y) +
        (sample - 1) * var(females_s$y))/(2 * sample - 2))
}

# glimpse(outdata)

ggplot(data = outdata, aes(x = diff, y = p)) + geom_point(aes(size = n),
    shape = 1) + theme_bw() + geom_hline(yintercept = 0.05, colour = "red",
    lty = 2) + geom_point(aes(x = mean(diff), y = 0.1), size = 3,
    colour = "blue") + geom_errorbarh(aes(y = 0.1, xmin = mean(diff) -
    sd(diff)/sqrt(nrow(outdata)), xmax = mean(diff) + sd(diff)/sqrt(nrow(outdata))),
    size = 0.2, height = 0.1, colour = "blue") + geom_point(aes(x = mean(filter(outdata,
    p < 0.05)$diff), y = 0.1), size = 3, colour = "purple") +
    geom_errorbarh(aes(y = 0.1, xmin = mean(filter(outdata, p <
        0.05)$diff) - sd(filter(outdata, p < 0.05)$diff)/sqrt(nrow(outdata)),
        xmax = mean(filter(outdata, p < 0.05)$diff) + sd(filter(outdata,
            p < 0.05)$diff)/sqrt(nrow(outdata))), size = 0.2,
        height = 0.1, colour = "purple") + geom_vline(xintercept = 10,
    colour = "red") + geom_text(aes(x = 10, y = 0.5, label = "true difference"),
    colour = "red", hjust = "left", nudge_x = 0.2) + xlab("Effect size (mean difference)") +
    ylab("p value")
```

### Figure S7

In this example, 26.6666667% of effect sizes would fail to satisfy
the 0.05 type-I error rate cut-off, and hence would be prone to being
omitted, potentially biasing the overall effect size (purple vs. true ES
in blue).

## Scenario 1 - low-powered studies are excluded

Based on significance, only “sexy” results are published. Example
meta-analysis shows, that this inflates the overall effect size.

```
# Calculate Cohen's d effect size
outdata <- outdata %>%
    mutate(d = diff/pooled_s, var_d = sqrt((2 * sample/(sample *
        sample)) + (d^2/(2 * (sample + sample)))))

model1 <- rma(yi = filter(outdata, p < 0.05)$d, vi = filter(outdata,
    p < 0.05)$var_d, method = "FE")
summary(model1)
```

```
## 
## Fixed-Effects Model (k = 4)
## 
##   logLik  deviance       AIC       BIC      AICc   
##  -2.4518    0.1816    6.9035    6.2898    8.9035   
## 
## I^2 (total heterogeneity / total variability):   0.00%
## H^2 (total variability / sampling variability):  0.06
## 
## Test for Heterogeneity:
## Q(df = 3) = 0.1816, p-val = 0.9805
## 
## Model Results:
## 
## estimate      se    zval    pval   ci.lb   ci.ub      
##   1.2821  0.3599  3.5624  0.0004  0.5767  1.9875  *** 
## 
## ---
## Signif. codes:  0 '***' 0.001 '**' 0.01 '*' 0.05 '.' 0.1 ' ' 1
```

```
forest(model1)
```

### Figure S8

A forest plot for Scenario 1

## Scenario 2 - conservative publication of all estimates

Publication mandate/archiving makes all effect sizes discoverable,
regardless of their significance and magnitude.

```
model2 <- rma(yi = outdata$d,
              vi = outdata$var_d
              #method = "FE"
              )
summary(model2)
```

```
## 
## Random-Effects Model (k = 15; tau^2 estimator: REML)
## 
##   logLik  deviance       AIC       BIC      AICc   
## -10.0477   20.0954   24.0954   25.3735   25.1863   
## 
## tau^2 (estimated amount of total heterogeneity): 0 (SE = 0.1875)
## tau (square root of estimated tau^2 value):      0
## I^2 (total heterogeneity / total variability):   0.00%
## H^2 (total variability / sampling variability):  1.00
## 
## Test for Heterogeneity:
## Q(df = 14) = 4.1608, p-val = 0.9944
## 
## Model Results:
## 
## estimate      se    zval    pval   ci.lb   ci.ub      
##   0.8628  0.1819  4.7430  <.0001  0.5063  1.2193  *** 
## 
## ---
## Signif. codes:  0 '***' 0.001 '**' 0.01 '*' 0.05 '.' 0.1 ' ' 1
```

```
forest(model2)
```

### Figure S9

A forest plot for Scenario 2

## If power calculation is based on meta-analytical effect-sizes

For biased (significant effect sizes only) overall estimate, the
simple power analysis suggests:

```
powercalc <- power.t.test(delta = summary(model1)$b, sig.level = 0.05,
    power = 0.8, sd = 1)
powercalc
```

```
## 
##      Two-sample t test power calculation 
## 
##               n = 10.60216
##           delta = 1.282074
##              sd = 1
##       sig.level = 0.05
##           power = 0.8
##     alternative = two.sided
## 
## NOTE: n is number in *each* group
```

In other words, it suggests sampling 11 individuals per group.

Achieving the same power assuming the conservative estimate of effect
size would require.

```
powercalc <- power.t.test(delta = summary(model2)$b, sig.level = 0.05,
    power = 0.8, sd = 1)
powercalc
```

```
## 
##      Two-sample t test power calculation 
## 
##               n = 22.08957
##           delta = 0.8628064
##              sd = 1
##       sig.level = 0.05
##           power = 0.8
##     alternative = two.sided
## 
## NOTE: n is number in *each* group
```

We need to sample 23 individuals per group (3 times larger N) Clearly
- the problem lies not in conducting under-powered studies, but in
*not publishing their results*. The degree of bias will be
exacerbated by having noisier data (larger sampling variance), low true
ES and clustering/non-additive effects modeled in addition.

# References

Anwer, H., Morris, M.J., Noble, D.W., Nakagawa, S. and Lagisz, M.,
2022. Transgenerational effects of obesogenic diets in rodents: A
meta‐analysis. Obesity Reviews, 23(1), p.e13342.

Gelman A, Carlin J. Beyond power calculations: Assessing type s
(sign) and type m (magnitude) errors. Perspectives on Psychological
Science. 2014;9: 641–651.

Lagisz M, Blair H, Kenyon P, Uller T, Raubenheimer D, Nakagawa S.
Transgenerational effects of caloric restriction on appetite: a
meta‐analysis. obesity reviews. 2014 Apr;15(4):294-309.

Lagisz, M., Blair, H., Kenyon, P., Uller, T., Raubenheimer, D. and
Nakagawa, S., 2015. Little appetite for obesity: meta-analysis of the
effects of maternal obesogenic diets on offspring food intake and body
mass in rodents. International Journal of Obesity, 39(12),
pp.1669-1678.

Lehr R. Sixteen s-squared over d-squared: A relation for crude sample
size estimates. Statistics in medicine. 1992;11: 1099–1102.

# R Session Information

```
sessionInfo() %>%
    pander()
```

**R version 4.2.1 (2022-06-23)**

**Platform:** x86\_64-apple-darwin17.0 (64-bit)

**locale:**
en\_AU.UTF-8||en\_AU.UTF-8||en\_AU.UTF-8||C||en\_AU.UTF-8||en\_AU.UTF-8

**attached base packages:** *stats*,
*graphics*, *grDevices*, *utils*,
*datasets*, *methods* and *base*

**other attached packages:** *metafor(v.3.8-1)*,
*metadat(v.1.2-0)*, *Matrix(v.1.5-3)*,
*pander(v.0.6.5)*, *Superpower(v.0.2.0)*,
*pwr(v.1.3-0)*, *retrodesign(v.0.1.0)*,
*readxl(v.1.4.1)*, *here(v.1.0.1)*,
*forcats(v.0.5.2)*, *purrr(v.1.0.0)*,
*readr(v.2.1.3)*, *tibble(v.3.1.8)*,
*tidyverse(v.1.3.2)*, *patchwork(v.1.1.1)*,
*cowplot(v.1.1.1)*, *ggplot2(v.3.4.0)*,
*stringr(v.1.5.0)*, *tidyr(v.1.2.1)*,
*magrittr(v.2.0.3)* and *dplyr(v.1.0.10)*

**loaded via a namespace (and not attached):**
*googledrive(v.2.0.0)*, *minqa(v.1.2.4)*,
*colorspace(v.2.0-3)*, *ellipsis(v.0.3.2)*,
*rprojroot(v.2.0.3)*, *estimability(v.1.4.1)*,
*fs(v.1.5.2)*, *rstudioapi(v.0.14)*,
*farver(v.2.1.1)*, *fansi(v.1.0.3)*,
*mvtnorm(v.1.1-3)*, *lubridate(v.1.8.0)*,
*mathjaxr(v.1.6-0)*, *xml2(v.1.3.3)*,
*codetools(v.0.2-18)*, *splines(v.4.2.1)*,
*cachem(v.1.0.6)*, *knitr(v.1.41)*,
*afex(v.1.2-1)*, *jsonlite(v.1.8.3)*,
*nloptr(v.2.0.3)*, *broom(v.1.0.2)*,
*dbplyr(v.2.2.1)*, *compiler(v.4.2.1)*,
*httr(v.1.4.4)*, *emmeans(v.1.8.2)*,
*backports(v.1.4.1)*, *assertthat(v.0.2.1)*,
*fastmap(v.1.1.0)*, *gargle(v.1.2.1)*,
*cli(v.3.4.1)*, *formatR(v.1.12)*,
*htmltools(v.0.5.3)*, *tools(v.4.2.1)*,
*lmerTest(v.3.1-3)*, *coda(v.0.19-4)*,
*gtable(v.0.3.1)*, *glue(v.1.6.2)*,
*reshape2(v.1.4.4)*, *Rcpp(v.1.0.8.3)*,
*carData(v.3.0-5)*, *cellranger(v.1.1.0)*,
*jquerylib(v.0.1.4)*, *vctrs(v.0.5.0)*,
*nlme(v.3.1-157)*, *xfun(v.0.34)*,
*lme4(v.1.1-30)*, *rvest(v.1.0.3)*,
*lifecycle(v.1.0.3)*, *pacman(v.0.5.1)*,
*googlesheets4(v.1.0.1)*, *MASS(v.7.3-58.1)*,
*scales(v.1.2.1)*, *hms(v.1.1.2)*,
*parallel(v.4.2.1)*, *yaml(v.2.3.6)*,
*sass(v.0.4.2)*, *stringi(v.1.7.8)*,
*highr(v.0.9)*, *boot(v.1.3-28)*, *rlang(v.1.0.6)*,
*pkgconfig(v.2.0.3)*, *evaluate(v.0.17)*,
*lattice(v.0.20-45)*, *labeling(v.0.4.2)*,
*tidyselect(v.1.2.0)*, *plyr(v.1.8.7)*,
*bookdown(v.0.31)*, *R6(v.2.5.1)*,
*generics(v.0.1.3)*, *DBI(v.1.1.3)*,
*pillar(v.1.8.1)*, *haven(v.2.5.1)*,
*withr(v.2.5.0)*, *abind(v.1.4-5)*,
*modelr(v.0.1.9)*, *crayon(v.1.5.2)*,
*car(v.3.1-0)*, *utf8(v.1.2.2)*, *tzdb(v.0.3.0)*,
*rmarkdown(v.2.17)*, *grid(v.4.2.1)*,
*rmdformats(v.1.0.4)*, *reprex(v.2.0.2)*,
*digest(v.0.6.30)*, *xtable(v.1.8-4)*,
*numDeriv(v.2016.8-1.1)*, *munsell(v.0.5.0)*,
*viridisLite(v.0.4.1)* and *bslib(v.0.4.0)*

LS0tCnRpdGxlOiAnRmluZGluZyB0aGUgcmlnaHQgcG93ZXIgYmFsYW5jZTogYmV0dGVyIHN0dWR5IGRlc2lnbiBhbmQgY29sbGFib3JhdGlvbiBjYW4gcmVkdWNlIGRlcGVuZGVuY2Ugb24gc3RhdGlzdGljYWwgcG93ZXInCnN1YnRpdGxlOiAiU2hpbmljaGkgTmFrYWdhd2EsIE1hbGdvcnphdGEgTGFnaXN6LCBZZWZlbmcgWWFuZyAmIFN6eW1vbiBEcm9ibmlhayIKZGF0ZTogICJgciBmb3JtYXQoU3lzLnRpbWUoKSwgJyVkICVCICVZJylgIgpvdXRwdXQ6IAogICAgCiAgICBybWRmb3JtYXRzOjpyZWFkdGhlZG93bjoKICAgICAgY29kZV9mb2xkaW5nOiBoaWRlCiAgICAgIGNvZGVfZG93bmxvYWQ6IHRydWUKZWRpdG9yX29wdGlvbnM6IAogIGNodW5rX291dHB1dF90eXBlOiBjb25zb2xlCiMgb3V0cHV0OgojICAgaHRtbF9kb2N1bWVudDoKIyAgICAgdG9jOiB5ZXMKIyAgICAgdG9jX2RlcHRoOiA0CiMgICAgIG51bWJlcl9zZWN0aW9uczogbm8KIyAgICAgdG9jX2Zsb2F0OgojICAgICAgIGNvbGxhcHNlZDogbm8KIyAgICAgICBzbW9vdGhfc2Nyb2xsOiBubwojICAgICBmaWcuYWxpZ246IGNlbnRlcgojICAgICBmaWdfY2FwdGlvbjogeWVzCiMgICAgIGVycm9yOiBubwojICAgICB3YXJuaW5nOiBubwojICAgICBtZXNzYWdlOiBubwojICAgICBlY2hvOiBubwojICAgICB0aWR5OiB5ZXMKIyAgICAgY2FjaGU6IHllcwojICAgICBkZl9wcmludDogcGFnZWQKIyAgICAgI2NvZGVfZm9sZGluZzogaGlkZQojICAgICB0aGVtZTogZmxhdGx5CiMgICB3b3JkX2RvY3VtZW50OgojICAgICB0b2M6IHllcwojICAgICB0b2NfZGVwdGg6IDQKIyAgIHBkZl9kb2N1bWVudDoKIyAgICAgdG9jOiB5ZXMKIyAgICAgdG9jX2RlcHRoOiA0CiNiaWJsaW9ncmFwaHk6IHJlZmVyZW5jZXMuYmliCiNjc2w6IHBsb3MuY3NsCmxpbmstY2l0YXRpb25zOiB5ZXMKLS0tCgoKYGBge3IsIGluY2x1ZGU9RkFMU0V9CiMjIyMjIyMjIyMjIyMjIyMjIyMjIyMjIyMjIyMjIyMjIyMjIyMjIyMjIyMjIyMjIyMjIyMjIyMjIyMjIyMjIyMjIyMjIyMjIyMjIwojIFN1cHBsZW1lbnRhcnkgTWF0ZXJpYWwgZm9yIFJpZ2h0IFBvd2VyIEJhbGFuY2UKIyBDb2RlIHdyaXR0ZW4gYnk6CiMgICAgICAgICBZZWZlbmcgWWFuZyAoeWVmZW5nLnlhbmcxQHVuc3cuZWR1LmF1KTsgU2Nob29sIG9mIEJpb2xvZ2ljYWwsIAojICAgICAgICAgICAgICAgICAgICAgICAgICAgICAgICAgICAgIEVhcnRoIGFuZCBFbnZpcm9ubWVudGFsIFNjaWVuY2VzLCAKIyAgICAgICAgICAgICAgICAgICAgIFVuaXZlcnNpdHkgb2YgTmV3IFNvdXRoIFdhbGVzLCBTeWRuZXksIEF1c3RyYWxpYSAgCiMgICAgICAgICBTaGluaWNoaSBOYWthZ2F3YSAocy5uYWthZ2F3YUB1bnN3LmVkdS5hdSk7IFNjaG9vbCBvZiBCaW9sb2dpY2FsLCAKIyAgICAgICAgICAgICAgICAgICAgICAgICAgICAgICAgICAgICBFYXJ0aCBhbmQgRW52aXJvbm1lbnRhbCBTY2llbmNlcywgCiMgICAgICAgICAgICAgICAgICAgICBVbml2ZXJzaXR5IG9mIE5ldyBTb3V0aCBXYWxlcywgU3lkbmV5LCBBdXN0cmFsaWEgIAojICAgICAgICAgU3p5bW9uIERyb2JuaWFrIChzLmRyb2JuaWFrQHVuc3cuZWR1LmF1KTsgU2Nob29sIG9mIEJpb2xvZ2ljYWwsIAojICAgICAgICAgICAgICAgICAgICAgICAgICAgICAgICAgICAgIEVhcnRoIGFuZCBFbnZpcm9ubWVudGFsIFNjaWVuY2VzLCAKIyAgICAgICAgICAgICAgICAgICAgIFVuaXZlcnNpdHkgb2YgTmV3IFNvdXRoIFdhbGVzLCBTeWRuZXksIEF1c3RyYWxpYSAgCiMjIyMjIyMjIyMjIyMjIyMjIyMjIyMjIyMjIyMjIyMjIyMjIyMjIyMjIyMjIyMjIyMjIyMjIyMjIyMjIyMjIyMjIyMjIyMjIyMjIyMgICAgICAgIApgYGAKCiMgRWxlY3Ryb25pYyBTdXBwbGVtZW50YXJ5IE1hdGVyaWFsCgojIFNldHVwcwoKYGBge3Igc2V0dXBzLCBlY2hvPUZBTFNFfQojIG92ZXJhbGwgUm1hcmtkb3duIHNldHRpbmdzCmtuaXRyOjpvcHRzX2NodW5rJHNldChpbmNsdWRlID0gVFJVRSwgbWVzc2FnZSA9IEZBTFNFLHdhcm5pbmcgPSBGQUxTRSwKICAgICAgICAgICAgICAgICAgICAgIHRpZHkub3B0cyA9IGxpc3Qod2lkdGguY3V0b2ZmID0gNjApLHRpZHkgPSBUUlVFLCAKICAgICAgICAgICAgICAgICAgICAgIGNhY2hlID0gVFJVRSwgZWNobz1UUlVFKQpgYGAKCkxvYWRpbmcgcGFja2FnZXMgYW5kIGN1c3RvbSBmdW5jdGlvbnMuIElmIHlvdXIgY29tcHV0ZXIgZG8gbm90IGhhdmUgdGhlIHJlcXVpcmVkIHBhY2thZ2VzLCBwbGVhc2UgaW5zdGFsbCB0aGVtIHZpYSBgaW5zdGFsbC5wYWNrYWdlcygicGFja2FnZS5uYW1lIilgCgpgYGB7ciBpbnN0YWxsaW5nfQojIGxvYWQgcmVxdWlyZWQgcGFja2FnZXMKcGFjbWFuOjpwX2xvYWQoZHBseXIsbWFncml0dHIsIHRpZHlyLCBzdHJpbmdyLCBnZ3Bsb3QyLCBjb3dwbG90LCBwYXRjaHdvcmssIHRpZHl2ZXJzZSwgaGVyZSwgcmVhZHhsLCByZXRyb2Rlc2lnbiwgcHdyLCBTdXBlcnBvd2VyLCBwYW5kZXIsIG1ldGFmb3IpCgojIGN1c3RvbSBmdW5jdGlvbiBmb3IgYXBwcm94aW1hdGUgc2FtcGxlIHNpemUgZm9yIG1haW4gZWZmZWN0IGFuZCBpbnRlcmFjdGl2ZSBlZmZlY3QKc2hvcnRfY3V0IDwtIGZ1bmN0aW9uKGQsIG1ldGhvZCA9IGMoIm5vcm1hbCIsICJpbnRlcmFjdGlvbiIpKXsKICBtZXRob2QgPC0gbWF0Y2guYXJnKG1ldGhvZCkKICBpZihtZXRob2QgPT0gIm5vcm1hbCIpewogICAgc2l6ZSA8LSAxNiooMS9kXjIpfQogIGVsc2V7CiAgICBzaXplIDwtIDMyKigxL2ReMikKICB9CiAgc2l6ZQp9CmBgYAoKIyBBaW1zIG9mIHRoaXMgU3VwcG9ydGluZyBJbmZvcm1hdGlvbgoKVGhlcmUgYXJlIHRocmVlIGRpZmZlcmVudCBzZWN0aW9ucyBpbiB0aGlzIGRvY3VtZW50OiAxKSB3ZSBwcmVzZW50cyBhbiBmaWN0aW9uYWwgc3RvcnkgdGhhdCBpbmNsdWRlIHNvbWUgb2YgZXhhbXBsZXMgb2Ygc21hbGwgZWZmZWN0cyBtYWlubHkgZm9jdXNpbmcgb24gaW50ZXJhY3Rpb24gZWZmZWN0cyB3ZSBtZW50aW9uIGluIHRoZSBtYWluIHRleHQgKEEgRmljdGlvbiksIDIpIHdlIHNob3cgaG93IHdlIG9idGFpbiBzYW1wbGUgc2l6ZXMgcHJlc2VudGVkIGluIHRoaXMgZmljdGl0aW91cyBzdG9yeSAoUG93ZXIgQ2FsY3VsYXRpb24pIGFuZCAzKSB3ZSBzaG93IHdoeSBpdCBpcyBpbXBvcnRhbnQgdG8gcHVibGlzaCByZWdhcmRsZXNzIG9mIHNhbXBsZSBzaXplcyBvciB0aGUgcG93ZXIgb2Ygc3R1ZGllcyAoU21hbGwgTiAmIE1ldGEtYW5hbHlzaXMpCgojIFNlY3Rpb24gMTogQSBGaWN0aW9uCgojIyBBIGxpa2VseSBzdG9yeSBvZiBhIHByaW5jaXBhbCBpbnZlc3RpZ2F0b3IKCkltYWdpbmUgdGhhdCB5b3UgYXJlIGEgbmV3IHByaW5jaXBhbCBpbnZlc3RpZ2F0b3IsIFBJLCBzdHVkeWluZyB0aGUgZGV0cmltZW50YWwgY29uc2VxdWVuY2VzIG9mIG9iZXNpdHkgb24gY29nbml0aW9uIHVzaW5nIGEgbW91c2UgbW9kZWwuIFlvdSBtdXN0IHB1dCB0b2dldGhlciB5b3VyIGZpcnN0IGdyYW50IGFzIGEgbGVhZCBQSS4gU28geW91IHN0YXJ0IHdpdGggcG93ZXIgYW5hbHlzaXMgdG8gZGV0ZXJtaW5lIHNhbXBsZSBzaXplIGZvciBhIG5ldyBpZGVhIG9uIGludmVzdGlnYXRpbmcgdGhlIGVmZmVjdCBvZiBtYXRlcm5hbCBvYmVzb2dlbmljIGRpZXQgb24gb2Zmc3ByaW5n4oCZcyBtZW1vcnkgdGFza3MgaW4gbWljZSBhbmQgc2V4LXNwZWNpZmljIGRpZmZlcmVuY2VzIGluIHRoZSB0YXNrcyAoRmlnIFMxQSkuIFlvdSBuZWVkIGFuIGVzdGltYXRlZCBlZmZlY3Qgb2YgdGhlIHRyZWF0bWVudCBhbmQgc2V4IGRpZmZlcmVuY2U7IG9mIGNvdXJzZSwgbm9ib2R5IGhhcyB5ZXQgcGVyZm9ybWVkIHN1Y2ggYSBzdHVkeSwgc28geW91IGRvIG5vdCBrbm93IGFuIGV4YWN0IGVmZmVjdCBzaXplLiBBbiBlYXJsaWVyIHN0dWR5IGV4YW1pbmluZyBtYXRlcm5hbCBvYmVzb2dlbmljIGRpZXQgb24gb2Zmc3ByaW5n4oCZcyBjb2duaXRpb24sIHVzaW5nIGEgZGlmZmVyZW50IGFzc2F5LCBoYXMgc3VnZ2VzdGVkIHN1cnByaXNpbmdseSBsYXJnZSBlZmZlY3RzOiBhIDMwJSBpbmNyZWFzZSBpbiB0aGUgbnVtYmVyIG9mIGVycm9ycyBpbiBtYWxlcyBhbmQgMjAlIGluIGZlbWFsZXMgKGkuZS4sIDEwJSBkaWZmZXJlbmNlIGJldHdlZW4gdGhlIHNleGVzOyBzdGFuZGFyZGl6ZWQgbWVhbiBkaWZmZXJlbmNlLCAkZCQgPSAxLjAsIDAuNjcsIDAuMzMsIGZvciBtYWxlIGFuZCBmZW1hbGUgdHJlYXRtZW50IGVmZmVjdHMsIGFuZCB0aGUgc2V4IGRpZmZlcmVuY2UsIHJlc3BlY3RpdmVseSkuIFVzaW5nIHRoZXNlIGVmZmVjdHMgc2l6ZXMsIHBvd2VyIGFuYWx5c2lzLCB3aGljaCBhc3N1bWVzIHRoZSBpbmRlcGVuZGVuY2Ugb2Ygc2FtcGxlcywgc2hvd3MgeW91IHdpbGwgbmVlZCB0aGUgc2FtcGxlIHNpemVzIG9mIDE2IG1hbGUgYW5kIDM2IGZlbWFsZSBtaWNlIChGMSBnZW5lcmF0aW9uKSBpbiBlYWNoIGNvbnRyb2wgYW5kIHRyZWF0bWVudCBncm91cHMgKDMyIG1hbGVzIGFuZCA3MiBmZW1hbGVzIGluIHRvdGFsKSB0byBkZXRlY3QgYSBzaW1pbGFyIGVmZmVjdCBpbiB5b3VyIHByb3Bvc2VkIGV4cGVyaW1lbnQuIFNvLCB5b3UgZGVjaWRlIHRoYXQgeW91IHdpbGwgdXNlIDcyIG9mZnNwcmluZyBtaWNlIGZvciBlYWNoIHNleCB0byBoYXZlIGEgbmljZWx5IGJhbGFuY2VkIGRlc2lnbiAoRmlnIFMxQikuIEJ1dCB0aGVuLCB5b3Ugd2lsbCBuZWVkIGF0IGxlYXN0IDM2IHggMiBtb3RoZXIgbWljZSAoRjAgZ2VuZXJhdGlvbikgY29uc2lkZXJpbmcgdGhlIGluZGVwZW5kZW5jZSBvZiBzYW1wbGVzIGF0IGxlYXN0IGluIHRyZWF0bWVudCB2cyBjb250cm9sIGdyb3VwcyAoaS5lLiwgdGFraW5nIG9uZSBtYWxlIGFuZCBvbmUgZmVtYWxlIHB1cCBmcm9tIGVhY2ggbW90aGVyIGZvciBlYWNoIGdyb3VwOyBzZWUgRmlnIFMxQykuCgohW10oLi4vZmlnL0ZpZ1MxLnBuZyl7d2lkdGg9NTAlfQoKIyMjIEZpZ3VyZSBTMQpBbiBleHBlcmltZW50YWwgZGVzaWduIHdpdGggaHlwb3RoZXRpY2FsIGVmZmVjdHMuIFBhbmVsIEEgc2hvd3MgYW4gb3ZlcnZpZXcgb2YgZXhwZXJpbWVudGFsIGRlc2lnbiB3aXRoIGZvdXIgZ3JvdXBzOiAxKSBjb250cm9sIGZlbWFsZXMgKE5jZiksIDIpIHRyZWF0bWVudCBmZW1hbGVzIChOb2YpLCAzKSBjb250cm9sIG1hbGVzIChOY20pIGFuZCA0KSB0cmVhdG1lbnQgbWFsZXMgKE5vbSkgd2l0aCBOIHJlcHJlc2VudGluZyDigJhzYW1wbGUgc2l6ZeKAmS4gUGFuZWwgQiBzaG93cyBhc3N1bWVkIGVmZmVjdCBzaXplcyBhcyAlIGFuZCBkIChzdGFuZGFyZGl6ZWQgbWVhbiBkaWZmZXJlbmNlOyBhc3N1bWluZywgZS5nLiB0aGUgYmFzZWxpbmUgbWVhbiBvZiAxMDAgYW5kIHN0YW5kYXJkIGRldmlhdGlvbiBvZiAyMCkgYW5kIHJlc3VsdGFudCBzYW1wbGUgc2l6ZXMgZnJvbSBwb3dlciBhbmFseXNpcy4gUGFuZWwgQyBzaG93cyB0d28gcG9zc2libGUgc2NlbmFyaW9zIGJhc2VkIG9uIHBvd2VyIGFuYWx5c2lzOiBGMCBhcmUgbW90aGVycywgRjEgYXJlIG9mZnNwcmluZyBmZW1hbGVzIChwaW5raXNoKSBhbmQgbWFsZXMgKG5hdnkpIHdpdGggYSB0YXJnZXQgc2FtcGxlIHNpemUgb2YgMzYgb2Zmc3ByaW5nIHBlciBzZXggcGVyIGdyb3VwLiAKCkFsdGhvdWdoIHRoaXMgcGxhbm5lZCBleHBlcmltZW50IGlzIGp1c3Qgb25lIHBhcnQgb2YgeW91ciBncmFudCBwcm9wb3NhbCwgaXQgaXMgYWxyZWFkeSBlYXRpbmcgdXAgdGhlIGJ1ZGdldCwgZXZlbiB3aXRob3V0IGNvbnNpZGVyaW5nIHNleCBkaWZmZXJlbmNlcy4gIFRodXMsIHlvdSBkZWNpZGUgdGhhdCBpdCBpcyBhY2NlcHRhYmxlIHRvIHRha2UgNCBtYWxlIGFuZCA0IGZlbWFsZSBwdXBzIGZyb20gZWFjaCBtb3VzZSBtb3RoZXIsIHNvIHlvdSBvbmx5IG5lZWQgOSB4IDIgKDE4KSBtb3RoZXJzIGluIHRvdGFsIChGaWcgUzFDKS4gQnV0IHlvdXIgZGVzaWduIG5vdyB2aW9sYXRlcyB0aGUgc3RhdGlzdGljYWwgYXNzdW1wdGlvbiBvZiBpbmRlcGVuZGVudCBzYW1wbGVzLCB1c2VkIGZvciB0aGUgcG93ZXIgYW5hbHlzaXMgKHNlZSBGaWcgMSkuIEJ5IHRoZSB3YXksIHdoYXQgeW91IGRlY2lkZSBub3QgdG8gbWVudGlvbiBpbiB5b3VyIGdyYW50IHByb3Bvc2FsIGlzIHRoYXQgdGhlIG9yaWdpbmFsIHBvd2VyIGFuYWx5c2lzIGFsc28gc3VnZ2VzdCB5b3Ugd291bGQgbmVlZCAyOTQgaW5kZXBlbmRlbnQgb2Zmc3ByaW5nIG9mIGVhY2ggc2V4IHBlciBncm91cCB0byBjb21wYXJlIHNleCBkaWZmZXJlbmNlIGluIHRyZWF0bWVudCBlZmZlY3QsIG9yIHN0YXRpc3RpY2FsbHkgc3BlYWtpbmcsIHRlc3RpbmcgZm9yIGFuIGludGVyYWN0aW9uIGFtb25nIHRoZSBmb3VyIGdyb3VwcyAoaS5lLiwgMSwxNzYgb2Zmc3ByaW5nIG1pY2UgZnJvbSAyOTQgeCAyIFs1ODhdIG1vdGhlcnM7IGZvciBkZXRhaWxzKS4gVGhpcyBkZWNpc2lvbiBvZiBjb25jZWFsbWVudCwgZGVzcGl0ZSB5b3VyIGludGVyZXN0IGluIHNleCBkaWZmZXJlbmNlLCBpcyBiZWNhdXNlIG5vdCBvbmx5IHdvdWxkIGluY29ycG9yYXRpbmcgc3VjaCBhIGRlc2lnbiBiZSBmYXIgdG9vIGV4cGVuc2l2ZSwgYnV0IGFsc28gcnVubmluZyBzdWNoIGFuIGV4cGVyaW1lbnQgd291bGQgYmUgdG9vIGxhYm9yIGludGVuc2l2ZSwgZXZlbiB3aXRoIGEgUGhEIHN0dWRlbnQgYW5kIHRlY2huaWNpYW4uIFN1cmVseSwgdGhlIGV0aGljcyBjb21taXR0ZWUgd291bGQgbm90IGxpa2Ugc3VjaCBhIGxhcmdlIG1pY2UgZXhwZXJpbWVudCBldmVuIGlmIHlvdSBnb3QgZnVuZGVkLiAgCgpOb3cgbGV04oCZcyB1c2Ugc21hbGxlciBhbmQgbW9yZSByZWFsaXN0aWMsIGFsYmVpdCwgaHlwb3RoZXRpY2FsLCBlc3RpbWF0ZXMgb2YgZWZmZWN0IHNpemVzOiA1JSwgMyUsIDIlIGRpZmZlcmVuY2UgKCRkJCA9IDAuMTYsIDAuMSBvciAwLjA2LCByZXNwZWN0aXZlbHkpIGZvciB0aGUgdHJlYXRtZW50IGVmZmVjdCBmb3IgbWFsZXMsIGZlbWFsZXMsIGFuZCB0aGUgc2V4IGRpZmZlcmVuY2UgKG5vdGUgdGhhdCByZXBsaWNhdGlvbiB3b3JrIHN1Z2dlc3RzIHRoZXNlIGh5cG90aGV0aWNhbCBlZmZlY3Qgc2l6ZXMgYXJlIHJlYXNvbmFibGUpLiBGb3IgdGhpcyBzY2VuYXJpbywgeW91IHdpbGwgbmVlZCA1NzQgeCAyICgxLDE0OCkgbWFsZXMsIDEsNjAwIHggMiAoMywyMDApIGZlbWFsZXMgYW5kIDcsMTI4IG9mZnNwcmluZyBtaWNlIGluIGVhY2ggb2YgdGhlIGNvbnRyb2wgYW5kIHRyZWF0bWVudCBncm91cHMgb2YgYm90aCBzZXhlcyAoaS5lLiwgMjgsNTEyIG1pY2UgaW4gdG90YWwpIHRvIHJlYWNoIHN0YXRpc3RpY2FsIHNpZ25pZmljYW5jZS4gWW91IG11c3Qgbm90IGZvcmdldCB0aGF0IHlvdSB3aWxsIGFsc28gbmVlZCA3LDEyOCB4IDIgKDE0LDI1NikgbW90aGVycyB0byBwcm9kdWNlIHRoZXNlIG9mZnNwcmluZyBtaWNlIChvbmx5IHVzaW5nIG9uZSBtYWxlIGFuZCBvbmUgZmVtYWxlIHB1cCBwZXIgbW90aGVyIHRvIG1lZXQgdGhlIGluZGVwZW5kZW5jZSBhc3N1bXB0aW9uKS4gVGhlcmUgaXMgbm8gd2F5IG9mIGRvaW5nIHN1Y2ggYSDigJhiaWdnZXItdGhhbi15b3VyLXVuaXZlcnNpdHktZmFjaWxpdHktY2FuLXRha2XigJkgZXhwZXJpbWVudCwgZXNwZWNpYWxseSBhcyBhIG5ldyBQSSAoYXQgbGVhc3QsIG5vdCBieSB5b3Vyc2VsZikuIEluIGZhY3QsIHRoZXJlIGlzIGxpdHRsZSBvciBubyBpbmNlbnRpdmUgd2hhdHNvZXZlciB0byBwcm92aWRlIHJlYWxpc3RpYyBlc3RpbWF0ZXMgb2Ygc2FtcGxlIHNpemVzIHdoZW4gZ3JhbnQgYm9kaWVzIHJlcXVlc3Qg4oCYdmFsdWUgZm9yIG1vbmV54oCZIGFuZCBldGhpY3MgY29tbWl0dGVlcyBhc2sgeW91IHRvIG1pbmltaXplIHRoZSB1c2Ugb2YgYW5pbWFscy4gSW5zdGVhZCwgc3VjaCB3ZWxsLWludGVuZGVkIHJlY29tbWVuZGF0aW9ucyBoYXZlIGJlZW4gaW5jZW50aXZpemluZyBzY2llbnRpc3RzIHRvIHVzZSB0aGUgc21hbGxlc3Qgc2FtcGxlIHNpemVzIHBvc3NpYmxlIGJhc2VkIG9uIGdyZWF0bHkgaW5mbGF0ZWQgcHVibGlzaGVkIGVmZmVjdCBzaXplcyAoc2VlIHRoZSBtYWluIHRleHQpLiBOb3RlIHRoaXMgZXhhbXBsZSB3YXMgaW5zcGlyZWQgYnkgb3VyIGVhcmxpZXIgbWV0YS1hbmFseXNlcyBvZiByZWxhdGVkIHN0dWRpZXMgKExhZ2lzeiBldCBhbC4gMjAxNCwgMjAxNTsgQW53ZXIgZXQgYWwuIDIwMjIpCgojIFNlY3Rpb24gMjogUG93ZXIgQ2FsY3VhdGlvbgoKIyMgUHJlYW1ibGVzCgpTdGF0aXN0aWNhbCBwb3dlciBhcmUgZGV0ZXJtaW5lZCBieSB0aGUgZm9sbG93aW5nIHRocmVlIHBhcmFtZXRlcnM6IAoKKDEpIFR5cGUgSSBlcnJvciBwcm9iYWJpbGl0eSwgJFxhbHBoYSQsIGFsc28ga25vd24gYXMgc2lnbmlmaWNhbmNlIHRocmVzaG9sZCwgd2hpY2ggaXMgdXN1YWxseSBmaXhlZCBhdCAwLjA1IChzZWUgVGFibGUgSSk7CgooMikgc2FtcGxlIHNpemUsICRuJCwgdGhhdCBpcyB0aGUgbnVtYmVyIG9mIHN1YmplY3RzIHJlcXVpcmVkIGZvciBhbiBleHBlcmltZW50CgooMykgc3RhbmRhcmRpemVkIGVmZmVjdCBzaXplLCAkRVtcdGhldGFdL1xzcXJ0e1ZhcltcdGhldGFdfSQsIHdoZXJlICRcdGhldGEkIGlzIHRoZSBlZmZlY3Qgc2l6ZSBvZiBpbnRlcmVzdCwgd2hpY2ggaXMgaW5kaWNhdGVkIGJ5IHRoZSByZWFsIGRpZmZlcmVuY2UgYmV0d2VlbiB0d28gZ3JvdXBzIChpbiBvdXIgY2FzZTogb2Jlc29nZW5pYyBkaWV0IHZzLiBjb250cm9sIGRpZXQpLCAkRVtcdGhldGFdJCBpcyB0aGUgcG9wdWxhdGlvbiBhdmVyYWdlL2V4cGVjdGF0aW9uLCBhbmQgJFZhcltcdGhldGFdJCBpcyB0aGUgcmVzcGVjdGl2ZSB2YXJpYW5jZTsgbm90ZSB0aGF0IHN0YW5kYXJkaXplZCBtZWFuIGRpZmZlcmVuY2UgJGQkIGlzIGFuIGV4YW1wbGUgb2YgYSBzdGFuZGFyZGl6ZWQgZWZmZWN0IHNpemUgKGZvciBtb3JlIG9uIGVmZmVjdCBzaXplLCBzZWUgYWxzbyBGaWcgMiBhbmQgQm94IDIpLiAKCmBgYHtyIGZpZy5oZWlnaHQ9IDV9CiMgZHJhd2luZyBGaWcgMQojIyMgUGFyYW1ldGVyIDE6IGFscGhhIGxldmVsIHZzLiBwb3dlcgoKIyMjIyBzZXQgYSByYW5nZSBvZiBhbHBoYSBsZXZlbHMgKDAuMDEgdG8gMC4xKQphbHBoYV9yYW5nZSA8LSBzZXEoMC4wMDEsMSxieT0wLjAxKQoKIyMjIyBjYWxjdWxhdGUgcG93ZXIgYXQgdGhlIHNldCBhbHBoYSBsZXZlbHMKI3VzaW5nIGEgbWVkaXVtIG1hZ25pdHVkZSBvZiBzdGFuZGFyZGl6ZWQgZWZmZWN0IHNpemUgMC41IAojd2l0aCBhIHN0YW5kYXJkIGRldmlhdGlvbiBvZiAwLjIgCnBvd2VyX3JhbmdlIDwtIHJldHJvX2Rlc2lnbihBID0gMC41LCBzID0gMC4yLCBhbHBoYSA9IGFscGhhX3JhbmdlKSAKCiMjIyMgY3JlYXRlIGEgZGF0YSBmcmFtZQpwb3dlcl92c19hbHBoYSA8LSBkYXRhLmZyYW1lKGFscGhhID0gYWxwaGFfcmFuZ2UsIHBvd2VyID0gcG93ZXJfcmFuZ2UkcG93ZXIpCgojIyMjIHBsb3QKcG93ZXJfdnNfYWxwaGFfcGxvdCA8LSBnZ3Bsb3QocG93ZXJfdnNfYWxwaGEpICsgZ2VvbV9saW5lKGFlcyh4PWFscGhhLHk9cG93ZXIpLHNob3cubGVnZW5kPUYpICsgCiAgc2NhbGVfeV9jb250aW51b3VzKGJyZWFrcyA9IHNlcSgwLCAxLCAwLjIpLCBsaW1pdHMgPSBjKDAsIDEpKSArCiAgZ2VvbV9obGluZSh5aW50ZXJjZXB0ID0gMC44LCBjb2xvdXIgPSAicmVkIikgKwogIGxhYnMoeD0iVHlwZSAxIGVycm9yIChhbHBoYSkiLCB5PSJTdGF0aXN0aWNhbCBwb3dlciIsIAogICAgICAgdGl0bGUgPSAiKEEpIGFscGhhIGxldmVsIHZzLiBwb3dlciIpICsgdGhlbWVfYncoKQoKCiMjIyBQYXJhbWV0ZXIgMjogbiB2cy4gcG93ZXIKCiMjIyMgc2V0IGEgcmFuZ2Ugb2YgbiAoMiB0byAxMDApCm5fcmFuZ2UgPC0gc2VxKDIsMTAwLGJ5PTIpCgojIyBjYWxjdWxhdGUgcG93ZXIgZm9yIGEgdHdvLXNhbXBsZSB0IHRlc3QgKHR3by1pbmRlcGVuZGVudC1zYW1wbGVzLWRlc2lnbikKICMgdXNpbmcgYSBtZWRpdW0gbWFnbml0dWRlIG9mIHN0YW5kYXJkaXplZCBlZmZlY3Qgc2l6ZSAwLjUgCnBvd2VyX3JhbmdlMiA8LSBwd3IudC50ZXN0KGQ9MC41LCBuPW5fcmFuZ2UsIHNpZy5sZXZlbD0wLjA1LAogICAgICAgICAgICAgICAgICAgICAgICAgICB0eXBlPSJ0d28uc2FtcGxlIiwgYWx0ZXJuYXRpdmU9InR3by5zaWRlZCIpCgojIyMjIGNyZWF0ZSBhIGRhdGFmcmFtZQpwb3dlcl92c19uIDwtIGRhdGEuZnJhbWUobiA9IG5fcmFuZ2UsIHBvd2VyID0gcG93ZXJfcmFuZ2UyJHBvd2VyKQoKIyMjIyBwbG90CnBvd2VyX3ZzX25fcGxvdCA8LSBnZ3Bsb3QocG93ZXJfdnNfbikgKwogIGdlb21fbGluZShhZXMoeD1uLHk9cG93ZXIpLHNob3cubGVnZW5kPUYpICsgCiAgc2NhbGVfeV9jb250aW51b3VzKGJyZWFrcyA9IHNlcSgwLCAxLCAwLjIpLCBsaW1pdHMgPSBjKDAsIDEpKSArCiAgZ2VvbV9obGluZSh5aW50ZXJjZXB0ID0gMC44LCBjb2xvdXIgPSAicmVkIikgKwogIGxhYnMoeD0iU2FtcGxlIHNpemUgKG4pIiwgeT0iU3RhdGlzdGljYWwgcG93ZXIiLCAKICAgICAgIHRpdGxlID0gIihCKSBuIHZzLiBwb3dlciIpICsgdGhlbWVfYncoKQoKIyMjIFBhcmFtZXRlciAzOiBlZmZlY3Qgc2l6ZSB2cy4gcG93ZXIKCiMjIyMgY3JlYXRlIGEgcGxhdXNpYmxlIHJhbmdlIG9mIHN0YW5kYXJkaXplZCBlZmZlY3Qgc2l6ZXMKZXNfcmFuZ2UgPC0gc2VxKDAuMDEsMS4wMSxieT0wLjAxKQoKIyMjIyBjYWxjdWxhdGUgcG93ZXIgd2l0aCBhbHBoYSAwLjA1CnBvd2VyX3JhbmdlMyA8LSByZXRyb2Rlc2lnbjo6cmV0cm9fZGVzaWduKEEgPSBlc19yYW5nZSwgcyA9IDAuMiwgYWxwaGEgPSAwLjA1KQoKIyMjIyBjcmVhdGUgYSBkYXRhZnJhbWUKcG93ZXJfdnNfZXMgPC0gZGF0YS5mcmFtZShlcyA9IGVzX3JhbmdlLCAKICAgICAgICAgICAgICAgICAgICAgICAgICBwb3dlciA9IHBvd2VyX3JhbmdlMyRwb3dlciwgYWxwaGEgPSByZXAoYygiMC4wNSIpLGxlbmd0aChlc19yYW5nZSkpKQoKIyMjIyBwbG90CnBvd2VyX3ZzX2VzX3Bsb3QgPC0gZ2dwbG90KHBvd2VyX3ZzX2VzKSArIAogIGdlb21fbGluZShhZXMoeD1lcyx5PXBvd2VyKSxzaG93LmxlZ2VuZD1GKSArIAogIHNjYWxlX3lfY29udGludW91cyhicmVha3MgPSBzZXEoMCwgMSwgMC4yKSwgbGltaXRzID0gYygwLCAxKSkgKwogIGdlb21faGxpbmUoeWludGVyY2VwdCA9IDAuOCwgY29sb3VyID0gInJlZCIpICsKICBsYWJzKHg9IkVmZmVjdCBzaXplIChkKSIsIHk9IlN0YXRpc3RpY2FsIHBvd2VyIiwgCiAgICAgICB0aXRsZSA9ICIoQykgZWZmZWN0IHNpemUgdnMuIHBvd2VyIikgKyAKICB0aGVtZV9idygpCgoKIyMjIHB1dCBhbGwgZmlndXJlcyB0b2dldGhlcgpwb3dlcl9wbG90IDwtIHBvd2VyX3ZzX2FscGhhX3Bsb3QgLyBwb3dlcl92c19uX3Bsb3QgLyBwb3dlcl92c19lc19wbG90IApwb3dlcl9wbG90CmBgYAoKIyMjIEZpZ3VyZSBTMgpBbiBleGFtcGxlIHNob3dpbmcgaG93IHRoZSB0aHJlZSBwYXJhbWV0ZXJzIGFmZmVjdCB0aGUgc3RhdGlzdGljYWwgcG93ZXI6IChBKSBUeXBlIEkgZXJyb3IgKCRcYWxwaGEkKSwgKEIpIFNhbXBsZSBzaXplICgkbiQpLCAoQykgTWFnbml0dWRlIG9mIHRoZSBzdGFuZGFyZGl6ZWQgZWZmZWN0IHNpemUgKCRkJCkuIFRoZXNlIGZpZ3VyZXMgYXJlIHNpbXVsYXRlZCB1c2luZyBgcmV0cm9fZGVzaWduKClgIGZ1bmN0aW9uIGluIGByZXRyb2Rlc2lnbmAgcGFja2FnZSAoR2VsbWFuICYgQ2FybGluIDIwMTQpLiBTZWUgdGhlIGNvcnJlc3BvbmRpbmcgY29kZSBjaHVuayBmb3IgZGV0YWlsZWQgY29kZS4KCkZyb20gRmlndXJlIFMyQSB3ZSBjYW4gc2VlIHRoYXQgd2hlbiBhbiBleHBlcmltZW50IGNvbW1pdHMgYSBoaWdoZXIgVHlwZSAxIGVycm9yICh3aGljaCB3ZSBkbyBub3Qgd2FudCksIGl0IGlzIGVhc2llciB0byBhY2hpZXZlIGEgZGVzaXJlZCBzdGF0aXN0aWNhbCBwb3dlciAoaS5lLiwgQ29oZW4ncyByZWNvbW1lbmRhdGlvbjogODBcJSBwb3dlcikuIEluY3JlYXNpbmcgc2FtcGxlIHNpemUgKCRuJCkgYW5kIG1hZ25pdHVkZSBvZiBzdGFuZGFyZGl6ZWQgZWZmZWN0IHNpemUgYXJlIGVmZmVjdGl2ZSB3YXlzIHRvIGluY3JlYXNlIHRoZSBzdGF0aXN0aWNhbCBwb3dlciBvZiBhIGdpdmVuIGV4cGVyaW1lbnQgKEZpZ3VyZSBTMkIgYW5kIFMyQykuIAoKIyMgRXN0aW1hdGluZyBzYW1wbGUgc2l6ZXMgaW4gdGhlICdmaWN0aXRpb3VzJyBleHBlcmltZW50IHdpdGggbGFyZ2UgZWZmZWN0cwoKV2hlbiBkZXNpZ25pbmcgYW4gJ2ZpY3RpdGlvdXMnIGRpZXQgZXhwZXJpbWVudCBpbiB5b3VyIGdyYW50IHByb3Bvc2FsLCBZb3UgY2hvb3NlIGEgY29tbW9uIHNpZ25pZmljYW5jZSB0aHJlc2hvbGQsICRcYWxwaGEkID0gMC4wNSBhbmQgdGhlIG5vbWluYWwgcG93ZXIgbGV2ZWwgb2YgODAlLiBCYXNlZCBvbiB5b3VyIHBpbG90IG9yIGV4dGVybmFsIGluZm9ybWF0aW9uIChlLmcuLCByZWxldmFudCBzdHVkaWVzIG9yIGEgbWV0YS1hbmFseXNpcyBvbiBtYXRlcm5hbCBlZmZlY3QpLCB5b3UgYXNzdW1lIG1hdGVybmFsIG9iZXNvZ2VuaWMgZGlldCB3aWxsIGxlYWQgdG8gYSAzMFwlIGluY3JlYXNlIGluIHRoZSBudW1iZXIgb2YgbWlzdGFrZXMgaW4gYSBtZW1vcnkgdGFzayBpbiBtYWxlcywgMjBcJSBpbiBmZW1hbGVzIGFuZCAxMFwlIGRpZmZlcmVuY2UgYmV0d2VlbiB0aGUgc2V4ZXMuIFRvIHF1YW50aWZ5IHRoZSBkaWV0IGVmZmVjdCB1c2luZyBhIHN0YW5kYXJkaXplZCBlZmZlY3Qgc2l6ZSAoaS5lLiwgJGQkKS4gV2UgYXNzdW1lZCB0aGUgZm9sbG93aW5nczoKCmNvbnRyb2wgZ3JvdXAgKGJvdGggbWFsZSBhbmQgZmVtYWxlKSAtIG1lYW4gPSAxMDAgKGFyYml0cmFyeSB1bml0KSBhbmQgc3RhbmRhcmQgZGV2aWF0aW9uIChzZCkgPSAzMDsKCm1hbGUgdHJlYXRtZW50IGdyb3VwIC0gbWVhbiA9IDEzMCBhbmQgc2QgPSAzMDsKCmZlbWFsZSB0cmVhdG1lbnQgZ3JvdXAgLSBtZWFuID0gMTIwIGFuZCBzZCA9IDMwLgoKTm90ZSB0aGF0IHdlIGFzc3VtZSB0aGUgaG9tb2dlbmVpdHkgb2YgdmFyaWFuY2VzIGFtb25nIGdyb3VwcyAoaS5lLiBzZCA9IDMwKS4KCmBgYHtyfQojIyBzY2VuYXJpbyAxOiBsYXJnZSBlZmZlY3RzCgojIyMgc2V0IHVwIGFuIGluZGVwZW5kZW50IGRlc2lnbgpkZXNpZ24gPC0gQU5PVkFfZGVzaWduKAogIGRlc2lnbiA9ICIyYioyYiIsICMgaW5kZXBlbmRlbnQgZGVzaWduLCB3aGljaCBtZWFucyBubyBjb3JyZWxhdGlvbgogIG4gPTI5MCwgIyB0aGUgc2FtcGxlIHNpemUgaW4gZWFjaCBncm91cCBmb3IgdGVzdGluZyBzZXggZGlmZmVyZW5jZQogIG11ID0gYygxMzAsIDEyMCwgMTAwLCAxMDApLCAKICBzZCA9IDMwLAogIGxhYmVsbmFtZXMgPSBjKCJkaWV0IiwgIm9iZXNvZ2VuaWMgIiwgImNvbnRyb2wgIiwgInNleCIsICJtYWxlIiwgImZlbWFsZSIpLAogIHBsb3QgPSBGQUxTRSkKCm1lYW5wbG90X2xhcmdlRVMgPC0gZGVzaWduJG1lYW5zcGxvdCArIGxhYnMoeCA9ICJHcm91cHMiLCB5ID0gIk1lYW4iKQpGaWd1cmVfUzMgPC0gbWVhbnBsb3RfbGFyZ2VFUwpGaWd1cmVfUzMKYGBgCgojIyMgRmlndXJlIFMzClZpc3VhbGl6YXRpb24gb2YgdGhlIGFzc3VtZWQgbWVhbnMgYW5kIHN0YW5kYXJkIGRldmlhdGlvbiAoc2QpIG9mIGVhY2ggZ3JvdXAsIHVzaW5nIHRoZSBwYWNrYWdlIGBTdXBlcnBvd2VyYC4gRXJyb3IgYmFycyByZXByZXNlbnQgc2QuIEhlcmUgd2UgYXNzdW1lIGVhY2ggZ3JvdXAgaXMgaW5kZXBlbmRlbnQgKG5vdGUgdGhhdCB0aGlzIGlzIG5vdCBxdWl0ZSB0cnVlIGlmIHdlIHRha2Ugb25lIG1hbGUgYW5kIG9uZSBmZW1hbGUgZnJvbSBvbmUgbW90aGVyIGJ1dCBmb3IgY29udmVuaWVuY2UsIGxldCdzIGFzc3VtZSB0aGlzKQoKIyMjIFByZXN1bWVkIGVmZmVjdCBzaXplcyAobGFyZ2UpCgpVc2luZyB0aGVzZSBtZWFucyBhbmQgc2RzLCB3ZSBoYXZlIHRoZSBmb2xsb3dpbmcgc3RhbmRhcmRpemVkIG1lYW4gZGlmZmVyZW5jZSAkZCQsIGNvcnJlc3BvbmRpbmcgXCUgZGlmZmVyZW5jZXM6CgooMSkgMS4wLCBjb3JyZXNwb25kaW5nIHRvIGEgMzBcJSBpbmNyZWFzZSBpbiB0aGUgbnVtYmVyIG9mIG1pc3Rha2VzIGluIGEgbWVtb3J5IHRhc2sgaW4gbWFsZXMgYWZ0ZXIgYSBkaWV0IGludGVydmVudGlvbjsKCigyKSAwLjY3LCBjb3JyZXNwb25kaW5nIHRvIGEgMjBcJSBpbmNyZWFzZSBpbiB0aGUgbnVtYmVyIG9mIG1pc3Rha2VzIGluIGEgbWVtb3J5IHRhc2sgaW4gZmVtYWxlcyBhZnRlciBhIGRpZXQgaW50ZXJ2ZW50aW9uOwoKKDMpIDAuMzMsIGNvcnJlc3BvbmRpbmcgdG8gYSAxMFwlIHNleCBkaWZmZXJlbmNlIG9yIGludGVyYWN0aW9uIGJldHdlZW4gZGlldCBhbmQgc2V4LiAKCllvdSBhcmUgcGxhbm5pbmcgdG8gdXNlIGEgdHlwaWNhbCB0d28tc2FtcGxlIHQtdGVzdCB0byBleGFtaW5lIHRoZSBzdGF0aXN0aWNhbCBzaWduaWZpY2FuY2Ugb2YgdGhlIGRpZXQgZWZmZWN0IGluIG1hbGVzIGFuZCBmZW1hbGVzLiBUaGVuIHlvdSBjYW4gYXBwcm94aW1hdGUgc2FtcGxlIHNpemUgcmVxdWlyZWQgZm9yIGVhY2ggZ3JvdXAgdXNpbmcgdGhlIGZvbGxvd2luZyBmb3JtdWxhIChMZWhyIDE5OTIpOiAKCiQkCm4gPSAxNiBcZnJhY3tWYXJbXHRoZXRhXX0ge0VbXHRoZXRhXV4yfSA9IFx0ZXh0ezE2fSBcZnJhY3sxfSB7ZF4yfQokJApIb3dldmVyLCBmb3IgdGhlIGxhc3QgZWZmZWN0IChpbnRlcmFjdGlvbiBlZmZlY3QpLCB0aGlzIHJlcXVpcmVzIGNvbXBhcmluZyA0IGdyb3VwcyBzbyB0aGF0IHRoaXMgZm9ybXVsYSBkb2VzIG5vdCB3b3JrLiBZZXQgeW91IGNvdWxkIHN0aWxsIHVzZSB0aGlzIGZvcm11bGEgYnkgcmVwbGFjaW5nIDE2IHdpdGggMzIgYXMgaW50ZXJhY3Rpb24gaW52b2x2ZXMgZm91ciBncm91cHMgcmF0aGVyIHRoYW4gdHdvLiAKCiMjIyBTYW1wbGUgc2l6ZSBjYWxjdWxhdGlvbiBmb3IgZGlldCBlZmZlY3RzCgpJbiB0aGUgZm9sbG93aW5nIHNlY3Rpb24sIHdlIHVzZSBvdXIgY3VzdG9tIGZ1bmN0aW9uIChiYXNlZCBvbiB0aGUgYWJvdmUgZm9ybXVsYSkKYW5kIG9uZSBleGlzdGluZyBSIHBhY2thZ2UgYHB3cmAgdG8gZXN0aW1hdGUgdGhlIHNhbXBsZSBzaXplIHVzZWQgaW4geW91ciBwcm9wb3NlZCBleHBlcmltZW50IHdpdGggZGlmZmVyZW50IHNjZW5hcmlvcyAoc2FtcGxlIHNpemUgbWVudGlvbmVkIGluIHRoZSBmaWN0aXRpb3VzIHN0b3J5IGluIHRoZSBtYWluIHRleHQpLgoKYGBge3J9CgojIENvaGVuJ3MgZCBtYWxlCigxMzAgLSAxMDApLzMwCgojIENvaGVuJ3MgZCBmZW1hbGUKKDEyMCAtIDEwMCkvMzAKCiMgdGhlIGZpcnN0IHNldCAoc3VycHJpc2luZyBsYXJnZSBlZmZlY3RzKQoKIyMgbWFsZSB0cmVhdG1lbnQgZWZmZWN0CnB3ci50LnRlc3QoZCA9IDEsIHNpZy5sZXZlbCA9IDAuMDUsIHBvd2VyID0gMC44LCAKICAgICAgICAgICB0eXBlID0gInR3by5zYW1wbGUiLCBhbHRlcm5hdGl2ZSA9ICJ0d28uc2lkZWQiKSAKIyBvdXIgcmVzdWx0IGZyb20gb3VyIGN1c3RvbSBmdW5jdGlvbiBpcyB2ZXJ5IGNsb3NlCnB3cl9pbmRlcGVuZGVudF9tX2QxIDwtIHNob3J0X2N1dChkID0gMSwgbWV0aG9kID0gIm5vcm1hbCIpCgojIyBmZW1hbGUgdHJlYXRtZW50IGVmZmVjdApwd3IudC50ZXN0KGQgPSAwLjY3LCBzaWcubGV2ZWwgPSAwLjA1LCBwb3dlciA9IDAuOCwgCiAgICAgICAgICAgdHlwZSA9ICJ0d28uc2FtcGxlIiwgYWx0ZXJuYXRpdmUgPSAidHdvLnNpZGVkIikKcHdyX2luZGVwZW5kZW50X2ZfZDAuNjcgPC0gc2hvcnRfY3V0KGQgPSAwLjY3LCBtZXRob2QgPSAibm9ybWFsIikKCmBgYAoKCiMjIyBTYW1wbGUgc2l6ZSBjYWxjdWxhdGlvbiBmb3Igc2V4IGRpZmZlcmVuY2UgKGludGVyYWN0aW9uKQoKV2UgY2Fubm90IHVzZSBgcHdyLnQudGVzdGAgZm9yIGdldHRpbmcgc2FtcGxlIHNpemUgZm9yIHRoZSBzZXggZGlmZmVyZW5jZS4gU28gZmlyc3Qgd2UgY2FuIHVzZSBvdXIgZm9ybXVsYSBhbmQgdGhlbiwgd2UgdXNlIHRoZSBwYWNrYWdlIGBTdXBlcnBvd2VyYCB0byBvYnRhaW4gYSBzaW11bGF0aW9uIGJhc2VkIHNhbXBsZSBzaXplLiAKCmBgYHtyfQojIENvaGVuJ3MgZCBzZXggZGlmZmVyZW5jZSAoaW50ZXJhY3Rpb24pCigoMTMwIC0gMTAwKSAtICgxMjAgLSAxMDApKS8zMAoKcHdyX2luZGVwZW5kZW50X2lfZDAuMzMgPC0gc2hvcnRfY3V0KGQgPSAwLjMzLCBtZXRob2QgPSAiaW50ZXJhY3Rpb24iKSAKcHdyX2luZGVwZW5kZW50X2lfZDAuMzMKYGBgCgpNYWluIHJlc3VsdHMgb2YgdGhlIG91dHB1dHMgb2YgdGhlIGBBTk9WQV9leGFjdGAgd2hlbiBkZXRlY3RpbmcgbGFyZ2UgZWZmZWN0czoKCmBgYHtyfQojIyBzY2VuYXJpbyAxOiBsYXJnZSBlZmZlY3RzCiMjIyBwZXJmb3JtIEFOT1ZBIGFuZCBjYWxjdWxhdGUgcG93ZXIgZm9yIGluIGRlcGVuZGVudCBkZXNpZ24KcG93ZXJfcmVzdWx0cyA8LSBBTk9WQV9leGFjdChkZXNpZ24sIGFscGhhX2xldmVsID0gMC4wNSwgdmVyYm9zZSA9IEZBTFNFKQoKcG93ZXJfcmVzdWx0cyRtYWluX3Jlc3VsdHMKYGBgCgoKU2ltdWxhdGlvbiAodXNpbmcgdGhlIGBBTk9WQV9leGFjdGAgZnVuY3Rpb24pIHNob3dzIHRoYXQgY29sbGVjdGluZyBkYXRhIGZyb20gJG4kID0gYHIgcm91bmQocHdyX2luZGVwZW5kZW50X2lfZDAuMzMsIDApYCBGMSBtaWNlIChwZXIgZ3JvdXApCmhhcyBgciByb3VuZChwb3dlcl9yZXN1bHRzJG1haW5fcmVzdWx0cyRwb3dlclszXSwwKWBcJSBwb3dlciBmb3IgdGhlIGludGVyYWN0aW9uIG9yIHNleCBkaWZmZXJlbmNlIChzZWUgY29kZSBjaHVuayBmb3IgUiBzeW50YXgpLgoKV2UgYWxzbyBwbG90IGEgcG93ZXIgY3VydmUgb3ZlciBhIHJhbmdlIG9mIHNhbXBsZSBzaXplcyAoRmlndXJlIFM0KSwgZnJvbSB3aGljaCB5b3UgY2FuIHZpc3VhbGx5IGV4cGxvcmUgd2hldGhlciB0aGUgZXhwZWN0ZWQgcG93ZXIgaXMgYWNoaWV2ZWQgZm9yIHRoZSBpbnRlcmFjdGlvbiAoYm90dG9tIHBhbmVsKSwgYW5kIGlmIHNvLCBhdCB3aGljaCBzYW1wbGUgc2l6ZS4KCgpgYGB7cn0KcGxvdC5wb3dlciA8LSBwbG90X3Bvd2VyKGRlc2lnbiwgbWluX24gPSA1LCBtYXhfbiA9IDMwMCwgCiAgICAgICAgICAgICAgICAgICAgICAgICBkZXNpcmVkX3Bvd2VyID0gODAsIHBsb3QgPSBGQUxTRSkKCnBsb3QucG93ZXIkcGxvdF9BTk9WQSArIGxhYnMoeCA9ICJTYW1wbGUgc2l6ZSBwZXIgZ3JvdXAiKQpgYGAKCiMjIyBGaWd1cmUgUzQKUG93ZXIgY3VydmVzIGZvciBsYXJnZSBpbnRlci1nZW5lcmF0aW9uYWwgZWZmZWN0IGluIGEgZGVwZW5kZW50IGRlc2lnbiAoZGlldCBhbmQgc2V4IGFyZSBtYW5pcHVsYXRlZCBiZXR3ZWVuIGFuaW1hbHMpLiBUb3AgcGFuZWwgPSB0aGUgbWFpbiBlZmZlY3QgLSBkaWV0OyBNaWRkbGUgcGFuZWwgPSB0aGUgbWFpbiBlZmZlY3QgLSBzZXg7IEJvdHRvbSBwYW5lbCA9IHRoZSBpbnRlcmFjdGl2ZSBlZmZlY3QsIHNleCBkaWZmZXJlbmNlLiBUaGUgb3JhbmdlIGhvcml6b250YWwgbGluZXMgZGVub3RlIHRoZSBleHBlY3RlZCBzdGF0aXN0aWNhbCBwb3dlciAoODBcJSkuIE5vdGUgdGhhdCB0aGUgbWFpbiBkaWV0IGVmZmVjdCBpcyBhbiBhdmVyYWdlIGVmZmVjdCBvdmVyIHRoZSB0d28gc2V4ZXMuIAoKQXMgeW91IHNlZSwgdGhlIHNpbXVsYXRpb24tYmFzZWQgbWV0aG9kIHN1Z2dlc3RzIHdlIG5lZWQgMjg0IHN1YmplY3RzIHRvIHJlYWNoIDgwXCUgdG8gZGV0ZWN0IHRoZSBpbnRlcmFjdGlvbiBlZmZlY3QsIHdoaWNoIGNvbmZpcm0gd2hhdCB3ZSBvYnRhaW5lZCBmb3JtIHRoZSBmb3JtdWxhIHdhcyBjbG9zZSBlbm91Z2guIAoKCiMjIEVzdGltYXRpbmcgc2FtcGxlIHNpemVzIGluIHRoZSAnZmljdGljaW91cycgZXhwZXJpbWVudCB3aXRoIHJlYWxzdGljIChzbWFsbCkgZWZmZWN0cwoKVGhpcyB0aW1lLCB3ZSBhc3N1bWUgbWF0ZXJuYWwgb2Jlc29nZW5pYyBkaWV0IHdpbGwgaGF2ZSBtb3JlIHJlYWxpc3RpYyBlZmZlY3RzIG9uIHB1cHMnIG1lbW9yeTogYSA1XCUgaW5jcmVhc2UgaW4gbWFsZXMsIDNcJSBpbiBmZW1hbGVzIGFuZCB0aHVzIDFcJSBkaWZmZXJlbmNlIGluIHRoZSBkaWV0IGVmZmVjdCBiZXR3ZWVuIHRoZSBzZXhlcyAoaW50ZXJhY3Rpb24pLiAKClRvIGNhbGN1bGF0ZSAkZCQsIHlvdSBhc3N1bWU6IAoKY29udHJvbCBncm91cCAoYm90aCBtYWxlIGFuZCBmZW1hbGUpIC0gbWVhbiA9IDEwMCAoYXJiaXRyYXJ5IHVuaXQpIGFuZCBzZCA9IDMwOwoKbWFsZSB0cmVhdG1lbnQgZ3JvdXAgLSBtZWFuID0gMTA1IGFuZCBzZCA9IDMwOwoKZmVtYWxlIHRyZWF0bWVudCBncm91cCAtIG1lYW4gPSAxMDMgYW5kIHNkID0gMzAuCgpgYGB7cn0KIyMgc2NlbmFyaW8gMjogc21hbGwgZWZmZWN0cwojIyMgc2V0IHVwIGFuIGluZGVwZW5kZW50IGRlc2lnbgpkZXNpZ24yIDwtIEFOT1ZBX2Rlc2lnbigKICBkZXNpZ24gPSAiMmIqMmIiLCAjIGluZGVwZW5kZW50IGRlc2lnbgogIG4gPTcxMjgsICMgdGhlIHNhbXBsZSBzaXplIHVzZWQgZm9yIHRlc3Rpbmcgc2V4IGRpZmZlcmVuY2UKICBtdSA9IGMoMTA1LCAxMDMsIDEwMCwgMTAwKSwgCiAgc2QgPSAzMCwKICBsYWJlbG5hbWVzID0gYygiZGlldCIsICJvYmVzb2dlbmljICIsICJjb250cm9sICIsICJzZXgiLCAibWFsZSIsICJmZW1hbGUiKSwKICBwbG90ID0gRkFMU0UpCgptZWFucGxvdF9zbWFsbEVTIDwtIGRlc2lnbjIkbWVhbnNwbG90ICsgCiAgbGFicyh4ID0gIkdyb3VwcyIsIHkgPSAiTWVhbiIsIHRpdGxlID0gIlJlYWxpc3RpYyBzbWFsbCBlZmZlY3QiKQptZWFucGxvdF9zbWFsbEVTCmBgYAoKIyMjIEZpZ3VyZSBTNQpWaXN1YWxpemF0aW9uIG9mIHRoZSBleHBlY3RlZCBtZWFucyBhbmQgc3RhbmRhcmQgZGV2aWF0aW9uIChzZCkgb2YgZWFjaCBncm91cCB1c2luZyB0aGUgcGFja2FnZSBgU3VwZXJwb3dlcmAgdW5kZXIgbW9yZSByZWFsaXN0aWMgc2NlbmFyaW9zLiBFcnJvciBiYXJzIHJlcHJlc2VudCBzZC4gSGVyZSB3ZSBhc3N1bWUgZWFjaCBncm91cCBpcyBpbmRlcGVuZGVudCAobm90ZSB0aGF0IHRoaXMgaXMgbm90IHF1aXRlIHRydWUgaWYgd2UgdGFrZSBvbmUgbWFsZSBhbmQgb25lIGZlbWFsZSBmcm9tIG9uZSBtb3RoZXIgYnV0IGZvciBjb252ZW5pZW5jZSwgbGV0J3MgYXNzdW1lIHRoaXMpLgoKIyMjIFByZXN1bWVkIGVmZmVjdCBzaXplcyAoc21hbGwpCgpBcyB3aXRoIHRoZSBhYm92ZSwgd2UgYXNzdW1lZCBhbGwgZ3JvdXBzIHNoYXJlIGEgY29tbW9uIHNkID0gMzAgKHBvcHVsYXRpb24gc3RhbmRhcmQgZGV2aWF0aW9uKS4gVGhlbiB5b3UgY2FuIG9idGFpbiB0aGUgZm9sbG93aW5nIHN0YW5kYXJkaXplZCBtZWFuIGRpZmZlcmVuY2UgJGQkOgoKKDEpIDAuMTYsIGNvcnJlc3BvbmRpbmcgdG8gNVwlIGluY3JlYXNlIGluIHRoZSBudW1iZXIgb2YgbWlzdGFrZXMgaW4gYSBtZW1vcnkgdGFzayBpbiBtYWxlcyBhZnRlciBhIGRpZXQgaW50ZXJ2ZW50aW9uOwoKKDIpIDAuMSwgY29ycmVzcG9uZGluZyB0byAzXCUgaW5jcmVhc2UgaW4gdGhlIG51bWJlciBvZiBtaXN0YWtlcyBpbiBhIG1lbW9yeSB0YXNrIGluIGZlbWFsZXMgYWZ0ZXIgYSBkaWV0IGludGVydmVudGlvbjsKCigzKSAwLjA2LCBjb3JyZXNwb25kaW5nIHRvIDJcJSBzZXggZGlmZmVyZW5jZSBvciBpbnRlcmFjdGlvbiBiZXR3ZWVuIGRpZXQgYW5kIHNleC4KCiMjIyBTYW1wbGUgc2l6ZSBjYWxjdWxhdGlvbiBmb3IgZGlldCBlZmZlY3RzCgpGb2xsb3dpbmcgc2ltaWxhciBwcm9jZWR1cmVzIGluIGVzdGltYXRpbmcgc2FtcGxlIHNpemVzIGZvciBsYXJnZSBlZmZlY3RzIChzZWUgYWJvdmUpLCB5b3UgY2FuIG9idGFpbiBzYW1wbGUgc2l6ZXMgd2l0aCB0aGVzZSBuZXcgcHJlc3VtZWQgZWZmZWN0IHNpemVzCgpgYGB7cn0KIyBDb2hlbidzIGQgbWFsZQooMTA1IC0gMTAwKS8zMAoKIyBDb2hlbidzIGQgZmVtYWxlCigxMDMgLSAxMDApLzMwCgojIHRoZSBmaXJzdCBzZXQgKHJlYWxpc3RpYyBzbWFsbCBlZmZlY3RzKQoKIyMgbWFsZSB0cmVhdG1lbnQgZWZmZWN0CnB3ci50LnRlc3QoZCA9IDAuMTY3LCBzaWcubGV2ZWwgPSAwLjA1LCBwb3dlciA9IDAuOCwgCiAgICAgICAgICAgdHlwZSA9ICJ0d28uc2FtcGxlIiwgYWx0ZXJuYXRpdmUgPSAidHdvLnNpZGVkIikKcHdyX2luZGVwZW5kZW50X21fZDAuMTY3IDwtIHNob3J0X2N1dChkID0gMC4xNjcsIG1ldGhvZCA9ICJub3JtYWwiKQoKIyMgZmVtYWxlIHRyZWF0bWVudCBlZmZlY3QKcHdyLnQudGVzdChkID0gMC4xLCBzaWcubGV2ZWwgPSAwLjA1LCBwb3dlciA9IDAuOCwgCiAgICAgICAgICAgdHlwZSA9ICJ0d28uc2FtcGxlIiwgYWx0ZXJuYXRpdmUgPSAidHdvLnNpZGVkIikKcHdyX2luZGVwZW5kZW50X2ZfZDAuMSA8LSBzaG9ydF9jdXQoZCA9IDAuMSwgbWV0aG9kID0gIm5vcm1hbCIpCgpgYGAKCiMjIyBTYW1wbGUgc2l6ZSBjYWxjdWxhdGlvbiBmb3Igc2V4IGRpZmZlcmVuY2UgKGludGVyYWN0aW9uKQoKV2UgY2FuIGFsc28gdXNlIG91ciBmb3JtdWxhIHRvIGVzdGltYXRlIChhc3N1bWluZyBpbmRlcGVuZGVuY2Ugb2YgYWxsIGdyb3VwcykgCgpgYGB7cn0KIyBDb2hlbidzIGQgc2V4IGRpZmZlcmVuY2UgKGludGVyYWN0aW9uKQooKDEwNSAtIDEwMCkgLSAoMTAyIC0gMTAwKSkvMzAKCiMgc2V4IGRpZmZlcmVuY2UKcHdyX2luZGVwZW5kZW50X2lfZDAuMDY3IDwtIHNob3J0X2N1dChkID0gMC4wNjcsIG1ldGhvZCA9ICJpbnRlcmFjdGlvbiIpCnB3cl9pbmRlcGVuZGVudF9pX2QwLjA2NwpgYGAKCldlIHVzZSBhIHNpbWlsYXIgc2ltdWxhdGlvbi1iYXNlZCBhcHByb2FjaCB0byBlbXBpcmljYWxseSBjYWxjdWxhdGUgcG93ZXIgZm9yIHRoZSBpbnRlcmFjdGlvbiBlZmZlY3QgdXNpbmcgYFN1cGVycG93ZXJgLiBNYWluIHJlc3VsdHMgb2YgdGhlIG91dHB1dHMgb2YgdGhlIGBBTk9WQV9leGFjdGAgd2hlbiBhc3N1bWluZyBzbWFsbCBlZmZlY3RzOgoKYGBge3J9CiMjIHNjZW5hcmlvIDI6IHNtYWxsIGVmZmVjdHMKIyMjIHBlcmZvcm0gQU5PVkEgYW5kIGNhbGN1bGF0ZSBwb3dlciBmb3IgaW4gZGVwZW5kZW50IGRlc2lnbgpwb3dlcl9yZXN1bHRzMiA8LSBBTk9WQV9leGFjdChkZXNpZ24yLCBhbHBoYV9sZXZlbCA9IDAuMDUsIHZlcmJvc2UgPSBGQUxTRSkKcG93ZXJfcmVzdWx0czIkbWFpbl9yZXN1bHRzCmBgYAoKU2ltdWxhdGlvbnMgKHVzaW5nIHRoZSA8Y29kZT5BTk9WQV9leGFjdDwvY29kZT4gZnVuY3Rpb24pIHNob3cgdGhhdCBjb2xsZWN0aW5nIGRhdGEgZnJvbSAkbiQgPSBgciByb3VuZChwd3JfaW5kZXBlbmRlbnRfaV9kMC4wNjcsIDApYCBGMSBtaWNlIChwZXIgZ3JvdXApCmhhcyBgciByb3VuZChwb3dlcl9yZXN1bHRzMiRtYWluX3Jlc3VsdHMkcG93ZXJbM10sMilgXCUgcG93ZXIgZm9yIHRoZSBpbnRlcmFjdGlvbiBvciBzZXggZGlmZmVyZW5jZS4gU28gdGhlIHNpbXVsYXRpb24gcmVzdWx0IHNlZW1zIHRvIGNhdGNoIHdpdGggdGhlIHNhbXBsZSBzaXplIGVzdGltYXRlZCBieSB0aGUgZm9ybXVsYS4gCgojIyBDb3JybGVhdGVkIHNhbXBsZXMgYW5kIHN0YXRpc3RpY2FsIHBvd2VyCgpBcyBtZW50aW9uZWQsIGNvcnJlbGF0ZWQgc2FtcGxlcyBjYW4gaW5jcmVhc2UgdGhlIHN0YXRpc3RpY2FsIHBvd2VyIG9mIGFuIGV4cGVyaW1lbnQgc28gdGhhdCB3ZSByZXF1aXJlIGZld2VyIHNhbXBsZXMuIEhlcmUsIHdlIGFzc3VtZSB0aGF0IHNpYmxpbmcgdHJhaXRzIGFyZSBjb3JyZWxhdGVkICgkciQgPSAwLjUpIHJlZ2FyZGxlc3Mgb2Ygc2V4LiBXZSBmaW5kICRuJCA9IDM1NjQgY2FuIHJlYWNoIHRoZSBleHBlY3RlZCBzdGF0aXN0aWNhbCBwb3dlciAoODBcJSkgZm9yIGludGVyYWN0aW9uIChpLmUuLCBzZXggZGlmZmVyZW5jZSkuIFRoaXMgbnVtYmVyICgzNTY3KSBjb3JyZXNwb25kcyB0byAKCmBgYHtyfQojIyBzY2VuYXJpbyAxOiBzbWFsbCBlZmZlY3RzCiMjIyBwZXJmb3JtIEFOT1ZBIGFuZCBjYWxjdWxhdGUgcG93ZXIgZm9yIGluIGluZGVwZW5kZW50IGRlc2lnbgojIyMgYXNzdW1pbmcgdGhlIHNpYmxpbmdzIGFyZSB2ZXJ5IHNpbWlsYXIgdG8gZWFjaCBvdGhlciAtIHIgPSAwLjUKZGVzaWduMyA8LSBBTk9WQV9kZXNpZ24oCiAgZGVzaWduID0gIjJ3KjJ3IiwgIyBkZXBlbmRlbnQgZGVzaWduIAogIG4gPTM1NjQsIAogIHIgPSAwLjUsCiAgbXUgPSBjKDEwNSwgMTAzLCAxMDAsIDEwMCksIAogIHNkID0gMzAsCiAgbGFiZWxuYW1lcyA9IGMoImRpZXQiLCAib2Jlc29nZW5pYyIsICJjb250cm9sIiwgInNleCIsICJtYWxlIiwgImZlbWFsZSIpLAogIHBsb3QgPSBGQUxTRSkKCnBvd2VyX3Jlc3VsdHMzIDwtIEFOT1ZBX2V4YWN0KGRlc2lnbjMsIGFscGhhX2xldmVsID0gMC4wNSwgdmVyYm9zZSA9IEZBTFNFKQpwb3dlcl9yZXN1bHRzMyRtYWluX3Jlc3VsdHMKYGBgCgpUaGlzIG51bWJlciAoMzU2NykgY29ycmVzcG9uZHMgdG8gdGhlIHZhbHVlIGNhbGN1bGF0ZWQgZnJvbSB0aGUgZm9sbG93aW5nIGZvcm11bGE6CgokJApuX3tpbnRlcmFjdGlvbn0gPSAgXGZyYWN7MzJ9IHtkXjJ9KDEgLSByKQokJAoKVXNpbmcgdGhpcyBmb3JtdWxhLCB3ZSBjYW4gYXNzdW1lIGEgbG93ZXIgY29ycmVsYXRpb24gKCRyJCA9IDAuMjUpIGFuZCB0aGVuLCB3ZSBmaW5kICRuJCA9IDUzNDYuIEFzIGJlZm9yZSwgd2UgY2FuIHZlcmlmeSB0aGlzLCB1c2luZyBgQU5PVkFfZGVzaWduYDoKCmBgYHtyfQojIyBzY2VuYXJpbyAyOiBzbWFsbCBlZmZlY3RzCiMjIyBwZXJmb3JtIEFOT1ZBIGFuZCBjYWxjdWxhdGUgcG93ZXIgZm9yIGluIGluZGVwZW5kZW50IGRlc2lnbgojIGFzc3VtaW5nIHRoZSBzaWJsaW5ncyBhcmUgdmVyeSBzaW1pbGFyIHRvIGVhY2ggb3RoZXIgLSByID0gMC4yNQpkZXNpZ240IDwtIEFOT1ZBX2Rlc2lnbigKICBkZXNpZ24gPSAiMncqMnciLCAgICMgZGVwZW5kZW50IGRlc2lnbiAKICBuID01MzQ2LCAKICByID0gMC4yNSwKICBtdSA9IGMoMTA1LCAxMDMsIDEwMCwgMTAwKSwgCiAgc2QgPSAzMCwKICBsYWJlbG5hbWVzID0gYygiZGlldCIsICJvYmVzb2dlbmljIiwgImNvbnRyb2wiLCAic2V4IiwgIm1hbGUiLCAiZmVtYWxlIiksCiAgcGxvdCA9IEZBTFNFKQoKcG93ZXJfcmVzdWx0czQgPC0gQU5PVkFfZXhhY3QoZGVzaWduNCwgYWxwaGFfbGV2ZWwgPSAwLjA1LCB2ZXJib3NlID0gRkFMU0UpCnBvd2VyX3Jlc3VsdHM0JG1haW5fcmVzdWx0cwpgYGAKCkFzIHlvdSBzZWUsIHdpdGggJG4kID0gNTM0Niwgd2UgaGF2ZSB+ODBcJSBwb3dlci4gV2Ugbm90ZSB0aGF0LCBhcyBtZW50aW9uIGluIHRoZSB0ZXh0LCBmb3IgbW9yZSBjb21wbGV4IGRlc2lnbnMgKGUuZy4gaW5jbHVkaW5nIGRpZmZlcmVudCBzdHJhaW5zIG9mIG1pY2UpLCB3ZSBjYW5ub3QgdXNlIHRoZSBmb3JtdWxhIG9yIHRoZSBmdW5jdGlvbnMgZm9ybSBgU3VwZXJwb3dlcmAuIFdlIG5lZWQgdG8gdXNlIG90aGVyIHNvZnR3YXJlIHBhY2thZ2VzIHdoaWNoIGNvdWxkIGFjY29tbW9kYXRlIHN1Y2ggZGVzaWduIGZlYXR1cmVzLiAKCiMgU2VjdGlvbiAzOiBTbWFsbCBOICYgTWV0YS1hbmFseXNpcwoKIyMgU2ltdWxhdGluZyB0aGUgcG9wdWxhdGlvbgoKVHdvIGdyb3VwcyBvZiBpbmRpdmlkdWFscywgZS5nLiBtYWxlcyBhbmQgZmVtYWxlcywgd2l0aCBtZWFucyBvZiAxMCBhbmQgMTIsIHJlc3BlY3RpdmVseS4gVmFyaWFuY2VzIGFyZSBlcXVhbCAoPSAxMDApLgoKYGBge3J9CnNldC5zZWVkKDc3NzcpCm1lYW5fbSA8LSAxMAptZWFuX2YgPC0gMjAKc2QgPC0gc3FydCgxMDApCk4gPC0gODg4CgptYWxlcyA8LSBybm9ybShOLCBtZWFuX20sIHNkKQpmZW1hbGVzIDwtIHJub3JtKE4sIG1lYW5fZiwgc2QpCgpwb3BkYXRhIDwtIGRhdGEuZnJhbWUoeSA9IGMobWFsZXMsIGZlbWFsZXMpLCBzZXggPSByZXAoYygibSIsICJmIiksIGVhY2ggPSBOKSkKYGBgCgpgYGB7cn0KZ2dwbG90KGRhdGEgPSBwb3BkYXRhKSArCiAgZ2VvbV9kZW5zaXR5KGFlcyh5ID0geSwgY29sb3VyID0gc2V4LCBmaWxsID0gc2V4KSwKICAgICAgICAgICAgICAgYWxwaGEgPSAwLjQsIGxpbmV3aWR0aCA9IDAuOCkgKwogIGdlb21fYm94cGxvdChhZXMoeCA9IDAsIHkgPSB5LCBncm91cCA9IHNleCwgY29sb3VyID0gc2V4KSwKICAgICAgICAgICAgICAgcG9zaXRpb24gPSAiZG9kZ2UyIiwgd2lkdGggPSAwLjAxLCBzaXplID0gMC44KSArCiAgeGxhYigiRGVuc2l0eSIpICsgeWxhYigiTWVhc3VyZWQgdHJhaXQiKSArCiAgdGhlbWVfYncoKSArIGNvb3JkX2ZsaXAoKQpgYGAKCiMjIyBGaWd1cmUgUzYKVmlzdWFsaXppbmcgdGhlIGRpZmZlcmVuY2UgYmV0d2VlbiBmZW1hbGVzIChmKSBhbmQgbWFsZXMgKG0pLgoKIyMgU2ltdWxhdGlvbiBvZiBtdWx0aXBsZSBzdHVkaWVzCgpTaW11bGF0ZSBNID0gMTUgaW5kZXBlbmRlbnQgc3R1ZGllcyB0aGF0IGxvb2sgYXQgdGhlIHBvcHVsYXRpb24sIHRlc3RpbmcgZm9yIGRpZmZlcmVuY2UgYmV0d2VlbiBtYWxlcyBhbmQgZmVtYWxlcy4gU3R1ZGllcyByYW5nZSBmcm9tIG4gPSA1IHRvIG4gPSAxMAoKYGBge3J9Ck0gPC0gMTUKc2FtcGxlcyA8LSA1OjEwCm91dGRhdGEgPC0gZGF0YS5mcmFtZShkaWZmID0gbnVtZXJpYygwKSwgdCA9IG51bWVyaWMoMCksIHAgPSBudW1lcmljKDApLCBuID0gbnVtZXJpYygwKSwgcG9vbGVkX3MgPSBudW1lcmljKDApKQoKZm9yKGkgaW4gMTpNKSB7CiAgc2FtcGxlIDwtIHNhbXBsZShzYW1wbGVzLCAxKQogIG1hbGVzX3MgPC0gc3Vic2V0KHBvcGRhdGEsIHNleCA9PSAibSIpW3NhbXBsZSgxOk4vMiwgc2l6ZSA9IHNhbXBsZSwgcmVwbGFjZSA9IEYpLF0KICBmZW1hbGVzX3MgPC0gc3Vic2V0KHBvcGRhdGEsIHNleCA9PSAiZiIpW3NhbXBsZSgxOk4vMiwgc2l6ZSA9IHNhbXBsZSwgcmVwbGFjZSA9IEYpLF0KICAKICBleHBkYXRhIDwtIHJiaW5kKG1hbGVzX3MsIGZlbWFsZXNfcykKICAKICB0ZXN0IDwtIHQudGVzdCh5IH4gc2V4LCBkYXRhID0gZXhwZGF0YSkKICBvdXRkYXRhW2ksICJkaWZmIl0gPC0gdGVzdCRlc3RpbWF0ZVsxXSAtIHRlc3QkZXN0aW1hdGVbMl0KICBvdXRkYXRhW2ksICJuIl0gPC0gc2FtcGxlCiAgb3V0ZGF0YVtpLCAidCJdIDwtIHRlc3Qkc3RhdGlzdGljCiAgb3V0ZGF0YVtpLCAicCJdIDwtIHRlc3QkcC52YWx1ZQogIG91dGRhdGFbaSwgInBvb2xlZF9zIl0gPC0gc3FydCgoKHNhbXBsZS0xKSp2YXIobWFsZXNfcyR5KSArIChzYW1wbGUtMSkqdmFyKGZlbWFsZXNfcyR5KSkvKDIqc2FtcGxlLTIpKQp9CgojZ2xpbXBzZShvdXRkYXRhKQoKZ2dwbG90KGRhdGEgPSBvdXRkYXRhLCBhZXMoeCA9IGRpZmYsIHkgPSBwKSkgKwogIGdlb21fcG9pbnQoYWVzKHNpemUgPSBuKSwgc2hhcGUgPSAxKSArIHRoZW1lX2J3KCkgKwogIGdlb21faGxpbmUoeWludGVyY2VwdCA9IDAuMDUsIGNvbG91ciA9ICJyZWQiLCBsdHkgPSAyKSArCiAgZ2VvbV9wb2ludChhZXMoeCA9IG1lYW4oZGlmZiksIHkgPSAwLjEpLCBzaXplID0gMywgY29sb3VyID0gImJsdWUiKSArCiAgZ2VvbV9lcnJvcmJhcmgoYWVzKHkgPSAwLjEsIHhtaW4gPSBtZWFuKGRpZmYpIC0gc2QoZGlmZikvc3FydChucm93KG91dGRhdGEpKSwKICAgICAgICAgICAgICAgICAgICAgeG1heCA9IG1lYW4oZGlmZikgKyBzZChkaWZmKS9zcXJ0KG5yb3cob3V0ZGF0YSkpKSwKICAgICAgICAgICAgICAgICBzaXplID0gMC4yLCBoZWlnaHQgPSAwLjEsIGNvbG91ciA9ICJibHVlIikgKwogIGdlb21fcG9pbnQoYWVzKHggPSBtZWFuKGZpbHRlcihvdXRkYXRhLCBwIDwgMC4wNSkkZGlmZiksIHkgPSAwLjEpLCBzaXplID0gMywgY29sb3VyID0gInB1cnBsZSIpICsKICBnZW9tX2Vycm9yYmFyaChhZXMoeSA9IDAuMSwgeG1pbiA9IG1lYW4oZmlsdGVyKG91dGRhdGEsIHAgPCAwLjA1KSRkaWZmKSAtIHNkKGZpbHRlcihvdXRkYXRhLCBwIDwgMC4wNSkkZGlmZikvc3FydChucm93KG91dGRhdGEpKSwKICAgICAgICAgICAgICAgICAgICAgeG1heCA9IG1lYW4oZmlsdGVyKG91dGRhdGEsIHAgPCAwLjA1KSRkaWZmKSArIHNkKGZpbHRlcihvdXRkYXRhLCBwIDwgMC4wNSkkZGlmZikvc3FydChucm93KG91dGRhdGEpKSksCiAgICAgICAgICAgICAgICAgc2l6ZSA9IDAuMiwgaGVpZ2h0ID0gMC4xLCBjb2xvdXIgPSAicHVycGxlIikgKwogIGdlb21fdmxpbmUoeGludGVyY2VwdCA9IDEwLCBjb2xvdXIgPSAicmVkIikgKyAKICBnZW9tX3RleHQoYWVzKHggPSAxMCwgeSA9IDAuNSwgbGFiZWwgPSAidHJ1ZSBkaWZmZXJlbmNlIiksIGNvbG91ciA9ICJyZWQiLCBoanVzdCA9ICJsZWZ0IiwgbnVkZ2VfeCA9IDAuMikgKwogIHhsYWIoIkVmZmVjdCBzaXplIChtZWFuIGRpZmZlcmVuY2UpIikgKyB5bGFiKCJwIHZhbHVlIikKYGBgCgojIyMgRmlndXJlIFM3CkluIHRoaXMgZXhhbXBsZSwgYHIgc3VtKG91dGRhdGEkcCA8IDAuMDUpL25yb3cob3V0ZGF0YSkqMTAwYFwlIG9mIGVmZmVjdCBzaXplcyB3b3VsZCBmYWlsIHRvIHNhdGlzZnkgdGhlIDAuMDUgdHlwZS1JIGVycm9yIHJhdGUgY3V0LW9mZiwgYW5kIGhlbmNlIHdvdWxkIGJlIHByb25lIHRvIGJlaW5nIG9taXR0ZWQsIHBvdGVudGlhbGx5IGJpYXNpbmcgdGhlIG92ZXJhbGwgZWZmZWN0IHNpemUgKHB1cnBsZSB2cy4gdHJ1ZSBFUyBpbiBibHVlKS4KCiMjIFNjZW5hcmlvIDEgLSBsb3ctcG93ZXJlZCBzdHVkaWVzIGFyZSBleGNsdWRlZAoKQmFzZWQgb24gc2lnbmlmaWNhbmNlLCBvbmx5ICJzZXh5IiByZXN1bHRzIGFyZSBwdWJsaXNoZWQuIEV4YW1wbGUgbWV0YS1hbmFseXNpcyBzaG93cywgdGhhdCB0aGlzIGluZmxhdGVzIHRoZSBvdmVyYWxsIGVmZmVjdCBzaXplLgoKYGBge3J9CgojIENhbGN1bGF0ZSBDb2hlbidzIGQgZWZmZWN0IHNpemUKb3V0ZGF0YSA8LSBvdXRkYXRhICU+JSBtdXRhdGUoZCA9IGRpZmYvcG9vbGVkX3MsCiAgICAgICAgICAgICAgICAgICAgICAgICAgICAgIHZhcl9kID0gc3FydCgoMipzYW1wbGUvKHNhbXBsZSpzYW1wbGUpKSsoZF4yLygyKihzYW1wbGUrc2FtcGxlKSkpKSkKCm1vZGVsMSA8LSBybWEoeWkgPSBmaWx0ZXIob3V0ZGF0YSwgcCA8IDAuMDUpJGQsCiAgICAgICAgICAgICAgdmkgPSBmaWx0ZXIob3V0ZGF0YSwgcCA8IDAuMDUpJHZhcl9kLAogICAgICAgICAgICAgIG1ldGhvZCA9ICJGRSIpCnN1bW1hcnkobW9kZWwxKQpmb3Jlc3QobW9kZWwxKQpgYGAKCiMjIyBGaWd1cmUgUzgKQSBmb3Jlc3QgcGxvdCBmb3IgU2NlbmFyaW8gMQoKIyMgU2NlbmFyaW8gMiAtIGNvbnNlcnZhdGl2ZSBwdWJsaWNhdGlvbiBvZiBhbGwgZXN0aW1hdGVzCgpQdWJsaWNhdGlvbiBtYW5kYXRlL2FyY2hpdmluZyBtYWtlcyBhbGwgZWZmZWN0IHNpemVzIGRpc2NvdmVyYWJsZSwgcmVnYXJkbGVzcyBvZiB0aGVpciBzaWduaWZpY2FuY2UgYW5kIG1hZ25pdHVkZS4KCgpgYGB7cn0KbW9kZWwyIDwtIHJtYSh5aSA9IG91dGRhdGEkZCwKICAgICAgICAgICAgICB2aSA9IG91dGRhdGEkdmFyX2QKICAgICAgICAgICAgICAjbWV0aG9kID0gIkZFIgogICAgICAgICAgICAgICkKc3VtbWFyeShtb2RlbDIpCmZvcmVzdChtb2RlbDIpCmBgYAoKIyMjIEZpZ3VyZSBTOQpBIGZvcmVzdCBwbG90IGZvciBTY2VuYXJpbyAyCgojIyBJZiBwb3dlciBjYWxjdWxhdGlvbiBpcyBiYXNlZCBvbiBtZXRhLWFuYWx5dGljYWwgZWZmZWN0LXNpemVzCgpGb3IgYmlhc2VkIChzaWduaWZpY2FudCBlZmZlY3Qgc2l6ZXMgb25seSkgb3ZlcmFsbCBlc3RpbWF0ZSwgdGhlIHNpbXBsZSBwb3dlciBhbmFseXNpcyBzdWdnZXN0czoKCmBgYHtyfQpwb3dlcmNhbGMgPC0gcG93ZXIudC50ZXN0KGRlbHRhID0gc3VtbWFyeShtb2RlbDEpJGIsCiAgICAgICAgICAgICAgICAgICAgICAgICAgc2lnLmxldmVsID0gMC4wNSwKICAgICAgICAgICAgICAgICAgICAgICAgICBwb3dlciA9IDAuOCwgc2QgPSAxKQpwb3dlcmNhbGMKYGBgCgpJbiBvdGhlciB3b3JkcywgaXQgc3VnZ2VzdHMgc2FtcGxpbmcgYHIgY2VpbGluZyhwb3dlcmNhbGMkbilgIGluZGl2aWR1YWxzIHBlciBncm91cC4KCkFjaGlldmluZyB0aGUgc2FtZSBwb3dlciBhc3N1bWluZyB0aGUgY29uc2VydmF0aXZlIGVzdGltYXRlIG9mIGVmZmVjdCBzaXplIHdvdWxkIHJlcXVpcmUuCgpgYGB7cn0KcG93ZXJjYWxjIDwtIHBvd2VyLnQudGVzdChkZWx0YSA9IHN1bW1hcnkobW9kZWwyKSRiLAogICAgICAgICAgICAgICAgICAgICAgICAgIHNpZy5sZXZlbCA9IDAuMDUsCiAgICAgICAgICAgICAgICAgICAgICAgICAgcG93ZXIgPSAwLjgsIHNkID0gMSkKcG93ZXJjYWxjCmBgYAoKV2UgbmVlZCB0byBzYW1wbGUgYHIgY2VpbGluZyhwb3dlcmNhbGMkbilgIGluZGl2aWR1YWxzIHBlciBncm91cCAoMyB0aW1lcyBsYXJnZXIgTikgQ2xlYXJseSAtIHRoZSBwcm9ibGVtIGxpZXMgbm90IGluIGNvbmR1Y3RpbmcgdW5kZXItcG93ZXJlZCBzdHVkaWVzLCBidXQgaW4gKm5vdCBwdWJsaXNoaW5nIHRoZWlyIHJlc3VsdHMqLiBUaGUgZGVncmVlIG9mIGJpYXMgd2lsbCBiZSBleGFjZXJiYXRlZCBieSBoYXZpbmcgbm9pc2llciBkYXRhIChsYXJnZXIgc2FtcGxpbmcgdmFyaWFuY2UpLCBsb3cgdHJ1ZSBFUyBhbmQgY2x1c3RlcmluZy9ub24tYWRkaXRpdmUgZWZmZWN0cyBtb2RlbGVkIGluIGFkZGl0aW9uLgoKIyBSZWZlcmVuY2VzCgpBbndlciwgSC4sIE1vcnJpcywgTS5KLiwgTm9ibGUsIEQuVy4sIE5ha2FnYXdhLCBTLiBhbmQgTGFnaXN6LCBNLiwgMjAyMi4gVHJhbnNnZW5lcmF0aW9uYWwgZWZmZWN0cyBvZiBvYmVzb2dlbmljIGRpZXRzIGluIHJvZGVudHM6IEEgbWV0YeKAkGFuYWx5c2lzLiBPYmVzaXR5IFJldmlld3MsIDIzKDEpLCBwLmUxMzM0Mi4KCkdlbG1hbiBBLCBDYXJsaW4gSi4gQmV5b25kIHBvd2VyIGNhbGN1bGF0aW9uczogQXNzZXNzaW5nIHR5cGUgcyAoc2lnbikgYW5kIHR5cGUgbSAobWFnbml0dWRlKSBlcnJvcnMuIFBlcnNwZWN0aXZlcyBvbiBQc3ljaG9sb2dpY2FsIFNjaWVuY2UuIDIwMTQ7OTogNjQx4oCTNjUxLgoKTGFnaXN6IE0sIEJsYWlyIEgsIEtlbnlvbiBQLCBVbGxlciBULCBSYXViZW5oZWltZXIgRCwgTmFrYWdhd2EgUy4gVHJhbnNnZW5lcmF0aW9uYWwgZWZmZWN0cyBvZiBjYWxvcmljIHJlc3RyaWN0aW9uIG9uIGFwcGV0aXRlOiBhIG1ldGHigJBhbmFseXNpcy4gb2Jlc2l0eSByZXZpZXdzLiAyMDE0IEFwcjsxNSg0KToyOTQtMzA5LgoKTGFnaXN6LCBNLiwgQmxhaXIsIEguLCBLZW55b24sIFAuLCBVbGxlciwgVC4sIFJhdWJlbmhlaW1lciwgRC4gYW5kIE5ha2FnYXdhLCBTLiwgMjAxNS4gTGl0dGxlIGFwcGV0aXRlIGZvciBvYmVzaXR5OiBtZXRhLWFuYWx5c2lzIG9mIHRoZSBlZmZlY3RzIG9mIG1hdGVybmFsIG9iZXNvZ2VuaWMgZGlldHMgb24gb2Zmc3ByaW5nIGZvb2QgaW50YWtlIGFuZCBib2R5IG1hc3MgaW4gcm9kZW50cy4gSW50ZXJuYXRpb25hbCBKb3VybmFsIG9mIE9iZXNpdHksIDM5KDEyKSwgcHAuMTY2OS0xNjc4LgoKTGVociBSLiBTaXh0ZWVuIHMtc3F1YXJlZCBvdmVyIGQtc3F1YXJlZDogQSByZWxhdGlvbiBmb3IgY3J1ZGUgc2FtcGxlIHNpemUgZXN0aW1hdGVzLiBTdGF0aXN0aWNzIGluIG1lZGljaW5lLiAxOTkyOzExOiAxMDk54oCTMTEwMi4KCiMgUiBTZXNzaW9uIEluZm9ybWF0aW9uCgpgYGB7cn0Kc2Vzc2lvbkluZm8oKSAlPiUgcGFuZGVyKCkKYGBgCgo=
